# The synthesis of fructose-based surfactants†

**DOI:** 10.1039/d4gc00399c

**Published:** 2024-03-13

**Authors:** Hung-Chien Lin, Marios Kidonakis, J. P. Kaniraj, Ihor Kholomieiev, Balint Fridrich, Marc C. A. Stuart, Adriaan J. Minnaard

**Affiliations:** a Stratingh Institute for Chemistry, University of Groningen Nijenborgh 7 9747 AG Groningen The Netherlands a.j.minnaard@rug.nl; b SustaCons Klauzal street 30 1072 Budapest Hungary; c Groningen Biomolecular Sciences and Biotechnology Institute, University of Groningen Nijenborgh 7 9747 AG Groningen The Netherlands

## Abstract

This study describes the synthesis of a new class of surfactants that is based on the bioderived building blocks fructose, fatty acid methyl esters (FAME), and hydroxy propionitrile (cyanoethanol, 3-HP). The synthesis is scalable, is carried out at ambient conditions, and does not require chromatography. The produced surfactants have excellent surfactant properties with critical micelle concentrations and Krafft points comparable to current glucose-based surfactants.

## Abstract 2

Fructose and fatty acid methyl esters are the basis for the preparation of a novel class of surfactants.

## Graphical abstract

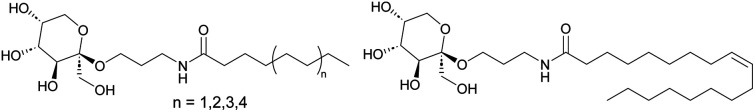

## Introduction

Surfactants are essential compounds in the chemical industry due to their application as detergents, softeners, cosmetics, coatings, and personal care products. With their amphiphilic character, surfactants tend to partition at interfaces with different degrees of polarity, such as water/oil, reducing the surface tension between the phases.^1^ Efficient surfactants can reduce the surface tension of air and water from 72 mN m^−1^ to 35 mN m^−1^, and the interfacial tension of n-hexadecane and water from 40 mN m^−1^ to 1 mN m^−1^.^2^

Most of the currently employed surfactants, such as alkyl- or alkylphenyl-based surfactants, are based on petrochemicals and are not sustainable.^3^ Furthermore, several surfactants harm living organisms,^4,5^ or degrade into toxic compounds that are persistent in the environment.^4,6^ To improve the sustainability of surfactants, the starting materials preferably are obtained from renewable sources. Furthermore, these surfactants should not be toxic, irritant,^7^ or hazardous to the environment and should degrade into non-toxic products.

## Surfactants based on carbohydrates

Carbohydrates undeniably are versatile building blocks for renewable surfactants.^8^ Commercially produced carbohydrate-based surfactants include sucrose esters, alkyl glucosides,^9^ and alkylpolyglycosides (APG).^10^ Whereas glycosylation invariably leads to an anomeric mixture of glycosides, sucrose esters, are obtained by transesterification with triglycerides in DMF at 120–130 °C. The drawback of this method is the required high temperature that leads to partial decomposition and coloration (browning) of the product which is an undesirable property in several applications including personal care.^11^ In addition, the use of DMF as a solvent is already discriminated by current regulations/policies and its replacement is imminent. DMF will be eliminated from many applications starting in 2024 due to strict EU regulations. Therefore, enzymatic reactions are studied to avoid these high temperatures.^12,13^ Fuglsang *et al.* combined enzymatic and chemical synthesis to produce sugar-ester cationic surfactants.^14^

Amino-sugars are interesting building blocks for the synthesis of surfactants as well, because amines are readily converted to amides, also in the presence of hydroxy groups. A procedure developed by Connor *et al.* describes the synthesis of fatty acid-based glucamides starting from glucose.^15^ RANEY® nickel-catalyzed reductive amination is followed by amidation with FAME to yield the corresponding glucamides. This approach only uses renewable materials, and the synthesis could be performed on a large scale. Very recently, the reductive amination of arabinose and galacturonic acid with long chain aliphatic amines has been reported to yield versatile surfactants.^16^

In a biocatalytic approach, Flitsch *et al.* implemented the enzyme carboxylic acid reductase to obtain methylglucamides by condensing methylglucamine 1 and fatty acids.^17^ This route is performed at ambient temperature in water and is in practice limited to shorter chain fatty acids. Column chromatography is used to remove salts from the product.

### Surfactants based on fructose

Next to glucose and sucrose,^18^ lactose,^19^ xylose,^20,21^ and furfural (a dehydrated sugar),^22^ have been explored for the synthesis of renewable surfactants. Surprisingly, there are only a handful of studies on the use of fructose in surfactant synthesis, although it is the second most abundant monosaccharide produced worldwide.^23^ Fischer glycosidation of fructose seems an attractive approach for the production of alkyl fructosides, but the glycosylation of fructose with higher alcohols at reflux triggers its dehydration, resulting in the formation of 5-hydroxymethylfurfural (HMF) and levulinic acid.^23^ Furthermore, Fischer glycosidation of fructose leads to a mixture of fructosides. To reduce side reactions in the synthesis of alkyl fructopyranosides, De Goede *et al.* used mesoporous MCM-41 acid catalysts with varying Si/Al ratios to catalyze the reactions at 80 °C and low pressure. The desired pyranosides were obtained by precipitation from the reaction mixture using diethyl ether, followed by recrystallization.^24^

As mentioned, Fischer glycosidation of fructose leads to a mixture of pyranosides and furanosides.^23,25^ Chittenden *et al.* prepared decyl fructofuranosides by reacting fructose and decanol with BF_3_–MeOH in ethanol.^26^ The use of pure decanol did not afford fructosides due to the low solubility of fructose. In order to obtain fatty fructopyranosides, Durbin *et al.* developed a glycosidation method using FeCl_3_ as a promoter to yield a series of alkyl fructopyranosides from fructose in fatty alcohols. The yields were around 30%.^27^ A different approach to prepare fructose-based surfactants has been reported by Lemaire *et al.*^28^ In their synthesis, the aldol-condensation of fructose with long chain aldehydes was performed. This method requires 20 eq. of fructose relative to the aldehyde, which necessitates an additional purification. Moderate yields were obtained with short-chain aldehydes whereas long-chain aldehydes led to low yields of the products.

We realized that a selective Fischer glycosylation of fructose, in combination with the use of fatty acids or their esters (in particular the FAME's that are readily available *via* non-energy intense routes), would provide a competitive route to renewable surfactants. Fatty esters are preferred over fatty alcohols as the latter are produced by hydrogenation of the former with a copper/chromium catalyst (copper chromite) at high temperatures and pressures (250–300 °C, and 250–300 bar H_2_).^29^ This is an energy costly process. We therefore re-evaluated an early finding (1985) by Chan *et al.*, who observed that Fischer glycosylation of fructose with chloroethanol led to selective formation of the β-fructopyranoside in an apparently dynamic crystallization process.^25^ Raaijmakers *et al.* elaborated on this finding and studied the scope in terms of suitable alcohols.^30,31^ The scope turned out to be broader than just chloroethanol but still limited, arguably because the product has to crystallize from the mixture.

**Fig. 1:**
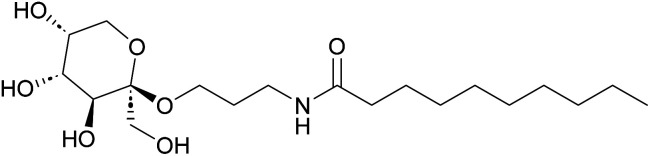
Example of a fructose-based surfactant prepared in this study.

**Scheme 1:**
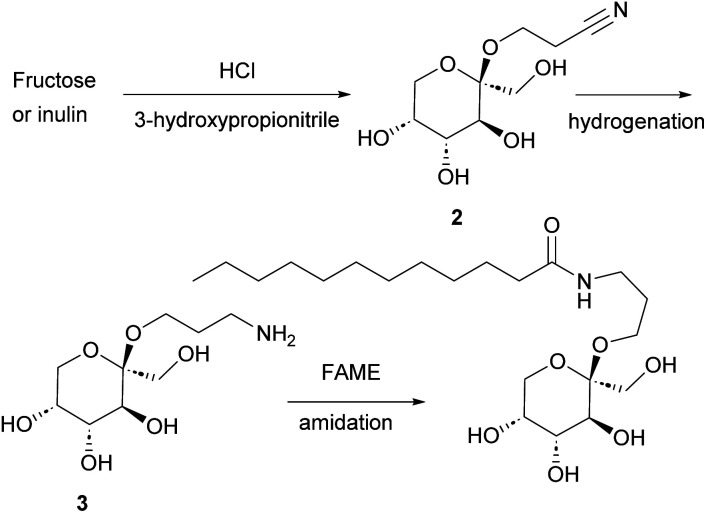
A synthetic route to fructose-based surfactants.

In the current study (Fig. 1 and Scheme 1) we report the selective Fischer glycosylation of fructose with hydroxypropionitrile (cyanoethanol, 3-HP). The nitrile function in this product is hydrogenated to the amine and subsequently subjected to base-catalyzed amidation with fatty acid methyl esters. This provides a novel class of fructose-based surfactants that shows desirable surfactant properties.

## Results and discussion

### Synthesis of cyanoethyl-β-fructopyranoside

Chan *et al.* observed that suspending d-fructose in acidified 2-chloroethanol led to selective crystallization of the corresponding β-fructopyranoside from the reaction mixture.^25^ Raaijmakers *et al.*^30^ expanded the scope of this reaction by reacting d-fructose with (next to 2-chloroethanol) 2-bromoethanol, 3-chloropropanol, and 4-chlorobutanol. In addition, sucrose and inulin were used as fructose source to yield the β-fructopyranosides. The reaction is efficient with chloro- and bromoethanol as well as 3-chloropropanol, but the yield dropped considerably when 4-chlorobutanol was used.^30^ The applicability of 2-butoxyethanol, 2-methoxyethanol, 2-(2-ethoxyethoxy)ethanol, 3-hydroxypropionitrile, 1-octanol, propargyl alcohol and 2,2,2-trichloroethanol was studied as well but these alcohols failed to yield the crystalline β-fructopyranoside. Allyl alcohol was used as well and provided the corresponding fructopyranoside in low yield.^31^

**Fig. 2:**
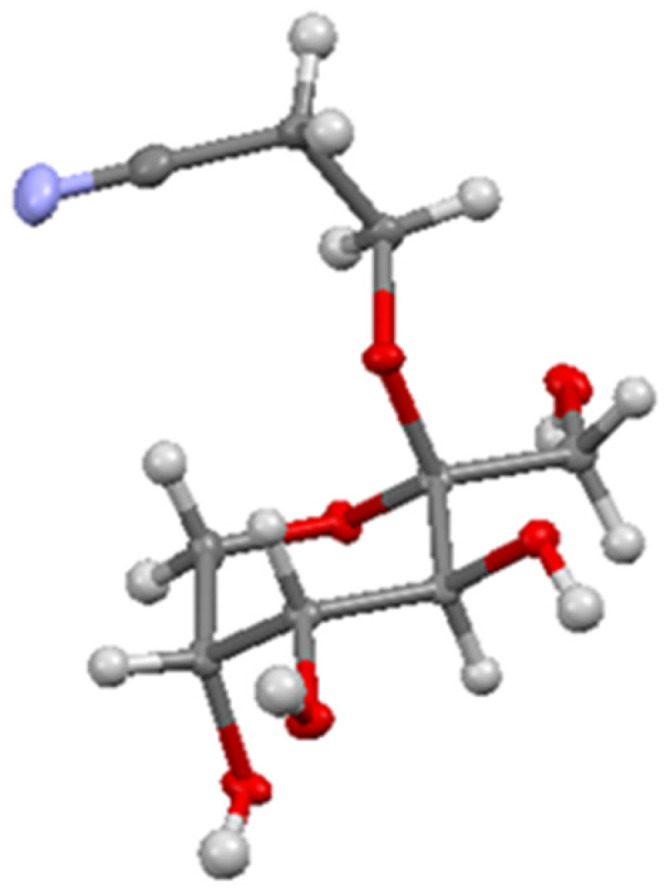
X-ray structure of cyanoethyl-β-fructopyranoside 2 (CCDC: 2310392†).

Fascinated by this crystallization-induced glycosidation reaction, we attempted to expand the scope with methanol, 2-methoxyethanol, ethylene glycol, and glycerol but all of these did not lead to the precipitation of the crystalline β-fructopyranoside. To our delight, however, we observed that 3-hydroxypropionitrile resulted in the formation of crystalline β-fructopyranoside 2 after slightly adjusting the reported conditions.^29^ β-fructopyranoside 2 was recrystallized and analyzed by X-ray diffraction (Fig. 2) thereby confirming the stereochemistry at the anomeric center.

3-Hydroxypropionitrile, a non-toxic compound, is currently industrially prepared by hydration of acrylonitrile. Bio-based acrylonitrile/3-hydroxypropionitrile is produced from glycerol or from glutamic acid.^32^ We therefore consider β-fructopyranoside 2 an interesting building block for novel bio-based surfactants. The nitrile group in 2 can be hydrogenated to an amine, followed by amidation with a fatty acid ester to yield surfactants. Therefore, we optimized the synthesis of β-fructopyranoside 2 from fructose, sucrose, and inulin.

### Synthesis of cyanoethyl-β-fructopyranoside 2 from d-fructose

Upon varying the fructose-hydroxypropionitrile ratio, precipitation of 2 started within 30 min after the start of the reaction, while the fructose was still dissolving. Usually, the entire reaction mixture did not turn homogeneous, so it was unclear whether all the fructose had been dissolved. Therefore, we performed the glycosidation reaction for 4 h. As expected, a higher yield of 2 was obtained when the reaction was performed at a higher fructose-hydroxypropionitrile ratio. On the other hand, the results with 0.5 and 1 g mL^−1^ at 5 g scale led to 68% and 61%, respectively (Table S1†). These results were considered comparable since the washing step in the filtration caused some experimental variation. On average, 2 was obtained in 60% yield. We found an optimum by using a slurry of 1 g mL^−1^ applying mechanical (overhead) stirring.

Subsequently, the concentration of acid was optimized, providing a balance between reaction speed, the need to remove the acid during the washing step, and potentially crystal size (Table S2†). As expected, smaller amounts of acid slowed down the reaction but the yield was unaffected. The crystal size did also not change to a notable extent. We settled therefore on a catalytic amount (6 mol%) of acid.

Upon scale-up, filtration of the product and subsequent washing became sluggish because of the small crystal size. After considerable experimentation, it was found that addition of isopropanol after the reaction followed by sonication of the reaction mixture, considerably improved filterability. In this way, batches of 250 g of fructose were routinely converted.

### Synthesis of cyanoethyl-β-fructopyranoside 2 from inulin and sucrose

Inulin is an oligo- or polysaccharide composed of fructofuranose units terminated by a glucose residue. Sucrose is a disaccharide composed of one glucose and one fructofuranose residue. Both are potential sources of fructose for the synthesis of 2, as already noted by Raaijmakers *et al.* for related compounds. With 1 mol% of acid, 0.25 g mL^−1^ hydroxypropionitrile, and ambient temperature, inulin produced 9% of 2 after 4 d and sucrose gave 24% of 2 mixed with glucose after 6 d. For comparison; fructose provided 38% of 2 under the same conditions in 4 h (Table S1†). The slower conversion is not unexpected since the fructofuranose form in both inulin and sucrose needs to transform into the fructopyranoside form in order to yield 2. This in contrast to crystalline fructose which is in the β-fructopyranose form. In addition, both glucose and sucrose have a very low solubility in hydroxypropionitrile.

Inulin has a fructose content of approximate 90%, and as expected, the yield of 2 improved by increasing the concentration of inulin to 0.5 (g mL^−1^), from 9% to 46%. Therefore, inulin is an acceptable starting material to prepare 2.

### Hydrogenation of cyanoethyl-β-fructopyranoside to aminoethyl-β-fructopyranoside 3

**Scheme 2:**
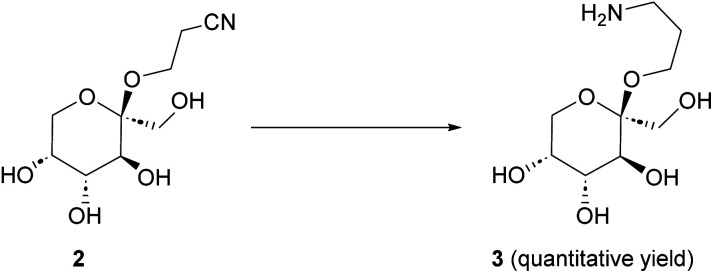
Hydrogenation of 2 to 3. (a) PtO_2_ (10 mol%), H_2_ (50 bar), 50 °C, 18 h. Or (b) RANEY® Nickel (15 wt%), NH_3_ (5 eq.), H_2_ (1 bar), 40 °C, 18 h.

Initial hydrogenation experiments (Scheme 2) were carried out using 10 mol% PtO_2_ (Adams’ catalyst), 50 bar H_2_, in MeOH at 50 °C.^33^ Full conversion of 2 to 3 was observed after 18 h. Reducing the catalyst loading to 5 mol%, however, only led to 50% conversion over 18 h. Therefore, other catalysts were considered. RANEY® Nickel (RaNi) is widely used in hydrogenation reactions and applied in various industrial processes due to its high reactivity and low(er) cost.^34,35^ To our delight, commercial aqueous RaNi suspension fully hydrogenated cyanoethyl-fructopyranoside 2 to aminoethyl-fructopyranoside 3 under ambient hydrogen atmosphere. We optimized this subsequently to 40 wt% of a 15 wt% aq. RaNi suspension, 5 eq. NH_3_, at 40 °C. Hydrogenation reactions at atmospheric pressure are, at least on lab scale, considerably more convenient. Therefore we selected RaNi as the hydrogenation catalyst of choice.

### Surfactants from aminoethyl-β-fructopyranoside 3

**Fig. 3:**
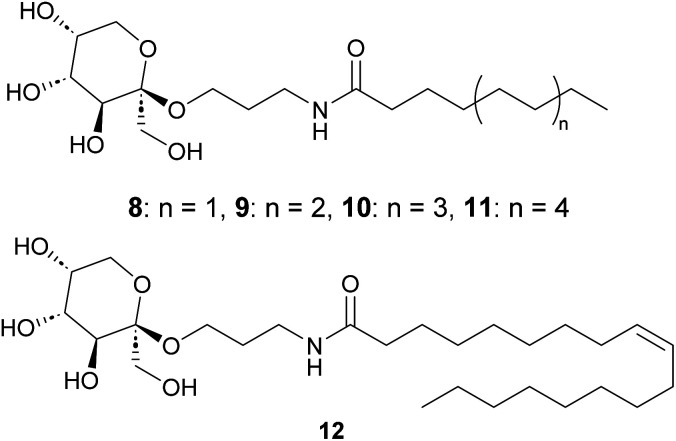
Fructose-based surfactants prepared according to the described method.

With 3 in hand, amidation with fatty acid methyl esters (FAME) should lead to the targeted surfactants. Connor and coworkers described the synthesis of glucamide surfactants by NaOMe-mediated amidation of methyl glucamine with FAME, using propylene glycol (1,2-propane diol) as the solvent at 85 °C.^15^ In addition, Esmaeili *et al.* used NaOMe-mediated amidation of α-alkylamino methyl esters to yield peptides.^36^ Therefore, we studied this reaction with amino-fructoside 3. The reaction was initially performed with 3 eq. FAME in the presence of 1 eq. NaOMe at 60 °C in methanol. Due to the hygroscopic nature of 3, and the use of an aqueous RaNi suspension in the preceding step, partial hydrolysis of the FAME initially was a competing reaction. This neutralized the NaOMe as well. Therefore, water had to be removed at <5 mbar and 50 °C, and the NaOMe was prepared from sodium and anhydrous methanol. At these optimized conditions, comprising 1.2 eq. of FAME and 1.5 eq. of NaOME at 50 °C, we obtained a series of fructose-based surfactants (Fig. 3). The overall yield of the synthesis ranges between 30% and 40%, not taking into account the possibility of recycling of the mother liquor in the first step. This is markedly lower than several reported yields in literature for carbohydrate-based surfactants, mainly because of a moderately-yielding precipitation of the final product.

### Properties of the fructose-based surfactants

With a series of fructose-based surfactants in hand, their physical properties were studied. The critical micelle concentration (CMC) is an important characteristic of surfactants since it indicates the minimum required amount of a surfactant to maximally reduce the surface tension of water.^37^ It should be noted, though, that the CMC is just one parameter that determines whether a surfactant is “fit for purpose” and we noted already the myriad of different applications of surfactants.

**Chart 1:**
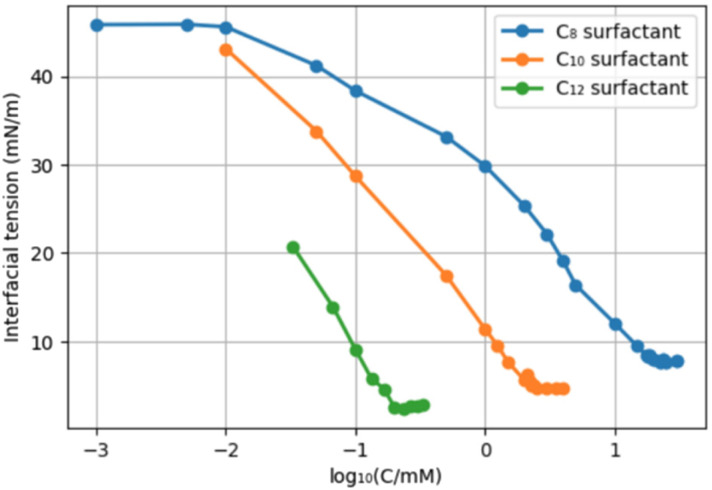
Surface tension *versus* surfactant concentration determined in a pentane/water system.

To perform CMC measurements, we utilized the pendant drop method in a water-pentane system. The reported interfacial tension of that system is 48.7 mN m^−1^ at 26 °C, and this value decreases with increasing temperature.^38^ According to our measurements at ambient temperature, the C_8_, C_10_, and C_12_-surfactants (8, 9, and 10) reached their CMC at 61.9, 6.4, and 0.6 mM, respectively (Chart 1). With 8, the interfacial tension was reduced to 7.7 mN m^−1^ at the CMC. For 9 and 10 this resulted in values of 4.7 and 2.4 mN m^−1^. These results show that the CMC reduces with increasing chain length (roughly one order of magnitude per “ethylene unit”), as expected, while surfactants with longer tails provide a somewhat lower minimal surface tension. Compounds 11 and 12 were only sparingly soluble in water at ambient temperature, so their CMC was not determined.

**Chart 2:**
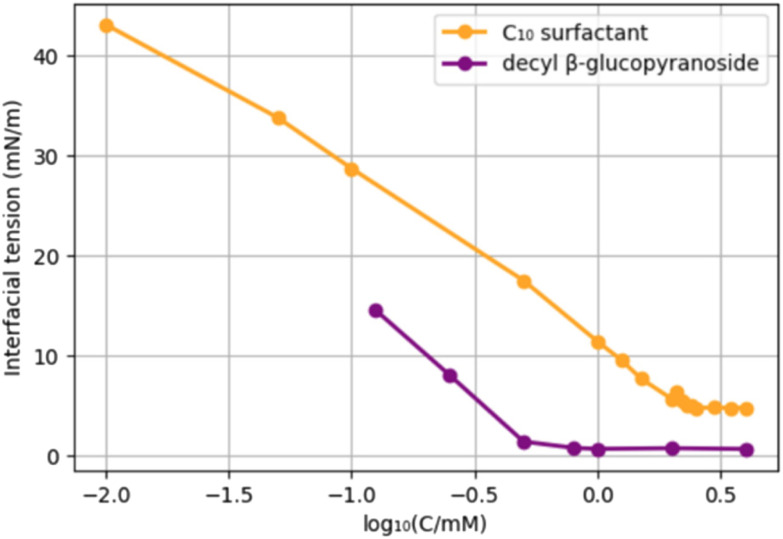
Comparison of the CMC of surfactant 9 (blue) with decyl glucopyranoside (orange).

In order to compare the new surfactants to existing surfactants, we selected decyl β-glucopyranoside, a C_10_-glucoside surfactant used as a solubilizer, and foaming detergent. An obvious difference with the newly prepared surfactants is the absence of an amide. We compared the CMC and interfacial tension of the C_10_-surfactant 9 with those of decyl glucopyranoside (Chart 2). The choice for 9 is somewhat arbitrary; although both surfactants have a C_10_ hydrocarbon chain, 9 has in addition a C_3_ “spacer” and an amide function. Decyl glucopyranoside provided a CMC of 2.5 mM (reported CMC = 2.2 mM),^39^ and an interfacial tension water/pentane of 0.7 mN m^−1^.^40^ C_10_-surfactant 9 has a CMC of 6.4 mM and a surface tension of 4.7 mN m^−1^. Potentially, the presence of an amide function leads to an increase in the CMC as well as the Krafft temperature, an effect that has been studied earlier.^41^

An alternative and straightforward method to determine the CMC of a surfactant is *via* the Nile Red CMC assay.^42^ This assay makes use of the change in fluorescence of the dye Nile Red upon incorporation in micelles. We used this assay (see ESI†) to determine the CMC of C_10_-surfactant 9, and obtained a CMC of 5.5–6 mM, which corresponds to the value measured with the pendant drop method (6.4 mM).

Although decyl glucopyranoside possesses a lower CMC, the difference is small. In general, an efficient surfactant can reduce the interfacial tension of water and *n*-hexadecane from 40 mN m^−1^ to 1 mN m^−1^.^42^ Our C_10_-surfactant 9 reduces the interfacial tension of water and *n*-pentane from 48.7 mN m^−1^ to 4.7 mN m^−1^, which is a similar span. Therefore, we conclude that, based on the CMC and the decrease in surface tension, fructose-hydroxypropionitrile-FAME-based 9 and its homologues are *bona fide* surfactants.

The so-called Krafft point is another important characteristic of surfactants. The Krafft point is defined as the intercept between the solubility curve and the temperature dependent CMC.^43^ This means that below the Krafft temperature, the solubility is lower than the CMC and the surfactant cannot form micelles. In general, surfactants with longer saturated alkyl chains give lower solubilities in water, and thus higher Krafft points.^44^ As micelles can only be formed above the Krafft point, surfactants are not very useful if the Krafft point is too high.

To determine the Krafft point, 0.2 wt% of a surfactant was solubilized in water followed by heating from ambient temperature at a rate of 1 °C min^−1^ until it entirely dissolved. Table S3† summarizes the characteristics of our developed surfactants.

According to the determined *T*_k_ (the approximate Krafft point) of the surfactants, the *T*_k_ increases with increasing chain length. The *T*_k_ of the C_10_ fructopyranoside 9 (36 °C) is similar but slightly higher than that of the C_10_-β-glucopyranoside (26 °C). The *T*_k_ of 11 and 12 are significantly higher, 75 °C and >90 °C, respectively. Compared to alkyl *N*-methyl glucamides, our surfactants have similar CMC, surface tension, and Krafft temperatures.^7,39,44,45^ Since amides provides additional hydrogen bonding interactions, it improves the packing between the molecules in the solid state, leading to higher Krafft temperatures than for alkyl glucosides.

**Fig. 4:**
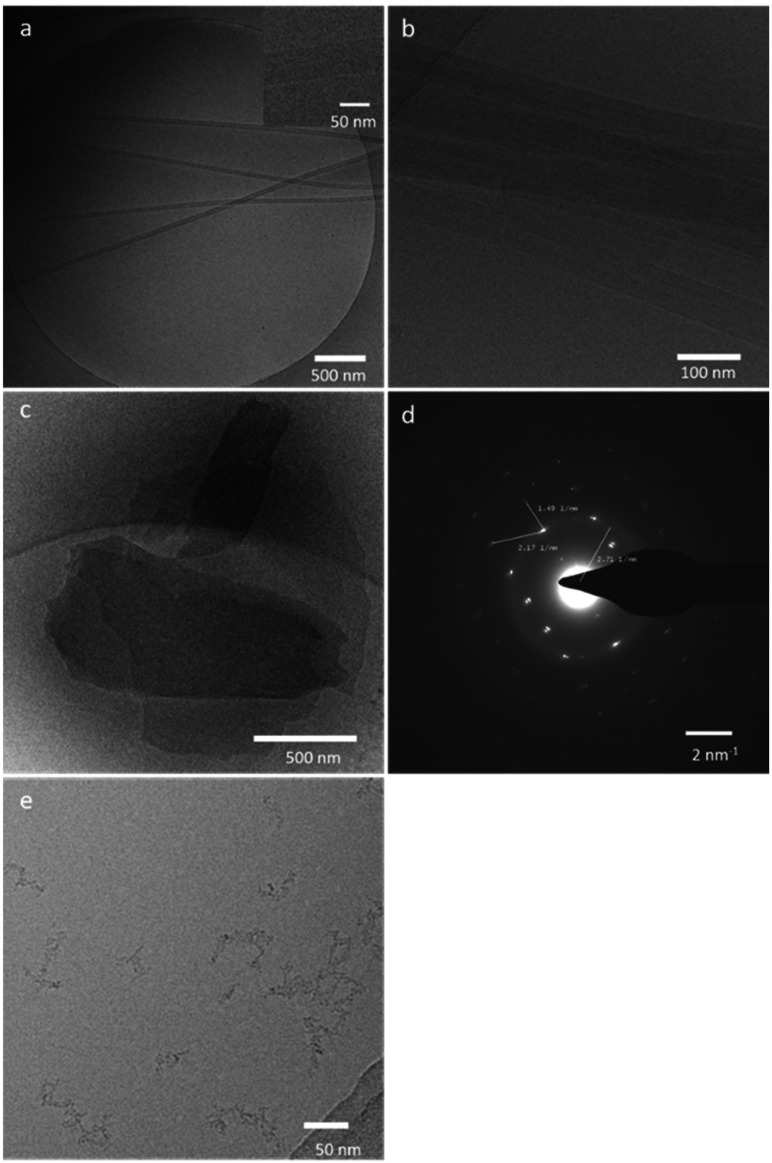
Cryo electron micrographs and a cryo-electron diffraction pattern of fructose-based surfactants. (a) Micrograph of sat. aqueous 8. (b) Micrograph of sat. aqueous 9. (c) Micrograph of sat. aqueous 10. (d) Diffraction pattern of the layered sheets formed from 10. (e) Formation of micelles from 10 above the Krafft temperature.

Below the Krafft temperature, surfactants generally form long crystalline fibers. The morphology of the fibers depends on the way the monomers aggregate.^46^ To get insight into the morphology of the fibers, we investigated an aqueous solution of 8 (>50 mg mL^−1^), 9 (10 mg mL^−1^), and 10 (2 mg mL^−1^) with cryo-electron microscopy. The non-homogeneous mixtures show two morphological differences. The shorter-chain surfactants, 8 and 9, formed lamellar fibers with the plane of the bilayers parallel to the fiber width (Fig. 4a and b). This aggregation pattern can be observed in the pictures by the lamellar striation on the fibers.^47^ Surfactant 8 tends to form infinitely long and ribbon-like fibers with a lamellar spacing of 3.3 nm (Fig. 4a). On the other hand, surfactant 9 shows short and stacked fibers with a bilayer spacing of 3.6 nm, indicating the morphology is slightly altered to layered aggregates (Fig. 4b). Interestingly, the aggregation of surfactant 10 is completely shifted to layered sheets (Fig. 4c). The lamellar striation cannot be observed from the surface of the sheets. This characteristic suggests that the lamellar organization of 10 is 90° rotated with respect to the fibers from the shorter-chain monomers. The plane of the bilayers is perpendicular to the width of the sheets. These results correspond to the morphological observation of sodium carboxylate fibers reported by Stuart *et al.*^48^ The cryo-electron diffraction pattern of the layered sheets of 10 clearly shows that the molecules are packed in a crystal-like manner with a spacing of 4.6 × 6.7 Å in the plane of the bilayers (Fig. 4d). We also observed a morphological change of 10-aggregates above the Krafft temperature. As expected, the sample of 10 turned clear upon heating, and the cryo-electron microscopy image showed the aggregated micelles (Fig. 4e).

Finally, we determined the foaming and emulsification behaviour of our surfactants. Foaming experiments of C_8_-8 and C_10_-9 were carried out according to literature^16^ and experimentally compared to the well-known surfactant SDS (see ESI†). An initial experiment demonstrated that for all three surfactants, the foam was stable for at least 10 min. This suggests that a commercial standard foam performance is achieved with this fructose-based surfactant. Subsequently, both the speed of foam formation and the foam stability were studied in more detail. It turned out that both 8, 9 and SDS have a very high speed of foam formation, and that the fructose-based surfactants 8 and 9 have a higher foam stability compared to SDS over the course of 1 h. Subsequent emulsification experiments with 9 showed that emulsification of sunflower oil is possible, but the resulting emulsion is not stable. This is congruent with the recently reported results of arabinose-based surfactants.^16^

## Conclusion

A series of fructose-based surfactants with C_8_- to C_18_-hydrocarbon chains (8–12) has been successfully prepared. The key step is the Fischer glycosylation of fructose, either as such or as constituent of inulin, with hydroxypropionitrile. Using fructose as a slurry in hydroxypropionitrile, the β-fructopyranoside form precipitates from the reaction mixture in a dynamic crystallization process. This avoids the selectivity problem normally encountered in the glycosylation of fructose and enables isolation of the product by filtration. Subsequent hydrogenation of the nitrile function to the amine and amidation with a variety of fatty acid methyl esters completes the surfactant synthesis. The synthesis is scalable and the utilized reagents are environmentally benign. Furthermore, no chromatography is required. With the abovementioned features, these fructose-based surfactants might be developed for industrial application. Determination of the CMC, Krafft point and foaming behaviour of the surfactants showed that these compare well to commercial non-ionic carbohydrate-based surfactants.

The results provide a solid basis to explore the scope of these fructose-based surfactants. Chain length and unsaturation pattern can be varied at will, providing tools to tailor the physical (surfactant) properties of the compounds. As the surfactants are pure, *e.g.* consist of one molecular entity, the experimental observations in terms of surfactant properties allow to be explained at the molecular level, and mixtures of surfactants of defined composition are readily prepared.

## Experimental section

### Solvents and reagents

All solvents and carbohydrates used were commercially available from Sigma-Aldrich, and used without further purification: decyl β-d-glucopyranoside, 3-hydroxy propionitrile, d-(−)-fructose, inulin (Carbosynth), sucrose, acetyl chloride, PtO_2_ (surface area ≥60 m^2^ g^−1^), 7 M ammonia in MeOH, Na, RANEY® Nickel (W.R. Grace and Co. RANEY®® 2800, slurry in water, active catalyst), methyl butyrate, methyl octanoate, methyl laurate, methyl palmitate, methyl oleate, Amberlyst® 15 hydrogen form, *N*,*O*-bis(trimethylsilyl)trifluoroacetamide, *N*-methyl-bis(trifluoroacetamide) (Sigma-Aldrich, for GC derivatization,^49,50^ Lichropur™, ≥97.0% (GC)).

### Analysis

^1^H-, ^13^C-, APT-, COSY-, HSQC- and HMBC-NMR were recorded on a Varian AMX400 spectrometer (400, 100 MHz, respectively) using DMSO-*d*_6_, or CD_3_OD as solvent. Chemical shift values are reported in ppm with the solvent resonance as the internal standard (DMSO-*d*_6_: *δ* 2.50 for ^1^H, *δ* 39.52 for ^13^C, CD_3_OD: *δ* 3.31 for ^1^H, *δ* 49.15 for ^13^C). Data are reported as follows: chemical shifts (*δ*), multiplicity (s = singlet, d = doublet, dd = double doublet, ddd = double double doublet, t = triplet, appt = apparent triplet, q = quartet, m = multiplet), coupling constants *J* (Hz), and integration. High-Resolution Mass spectrometry measurements were performed using a ThermoScientific LTQ OrbitrapXL spectrometer. Surface tension was measured using a Biolin Scientific optical tensiometer (Theta Lite).

### Synthesis of cyanoethyl-β-fructopyranoside 2

#### Cyanoethyl-β-fructopyranoside from fructose

250 ml of 3-hydroxypropionitrile and catalytic amounts of acetyl chloride were added to a 5 L round-bottom flask and mixed using an overhead stirrer for 15 min. 250 g of d-fructose was added to the flask and stirring was continued for 4 h. Part of the fructose gradually dissolved during the first 30 min, after which precipitation started and the mixture became progressively more viscous. Subsequently, 2 L of isopropanol was added and stirring was continued for 45 min in an ultrasonic bath. The obtained slurry was filtered (450 mbar, porosity 3 glass filter) and the cake was washed with isopropanol until no 3-hydroxypropionitrile was detected with ^1^H NMR. Drying *in vacuo* yielded 189 g (59%) of 2 as a white solid.

#### Cyanoethyl-β-fructopyranoside from inulin

In a 250 mL round bottom flask, 0.5 mL of acetyl chloride was added to 20 mL of 3-hydroxypropionitrile. The solution was stirred for 15 min at rt with an overhead stirrer. 10 g of inulin was added to the solution and left stirring at rt for 4 d. Subsequently, the resulting slurry was mixed with isopropanol to give a suspension. The suspended solid was collected by filtration with reduced pressure (using a fritted funnel with porosity 2 and 800 mbar). The collected solid was washed with isopropanol and dried under vacuum. 46% yield of 2 was obtained as a white solid.

### Synthesis of aminoethyl-β-fructopyranoside 3 by hydrogenation with RANEY® Nickel

To a 1 L three-necked flask, 8 gr of an aqueous RANEY®-Nickel suspension was added under nitrogen atmosphere. In order to remove the water, 20 mL of MeOH was added, the slurry was stirred for a few sec, stirring was halted and the MeOH was removed with a syringe. This procedure was repeated twice. At that point, 100 mL of MeOH was added, the system was evacuated and backfilled with nitrogen 3 times. A H_2_ balloon was attached, the RaNi/MeOH solution was flushed with hydrogen for around 5 to 10 min, and 20 g of 2 was added together with 100 mL of MeOH. Hydrogen was led through the solution for an additional 10 min. Subsequently, 5 eq. of NH_3_ in MeOH (7 M) was added and the system was heated to 40 °C overnight. Upon completion, stirring was terminated whereupon the RaNi stuck on the magnetic stirring bar. The mixture was passed through a pad of Celite, and the Celite was washed with MeOH. The material was collected in a one-neck flask. Note: the amine 3 is hydroscopic and should be stored under inert atmosphere.

### Surfactant synthesis by NaOMe mediated amidation of 3

Compound 3 from the previous step was co-evaporated with MeOH to remove residual water. The material appeared as a white foam, highly hydroscopic. 50 mL Of anhydrous MeOH was added and the solution was stirred for a few min to dissolve the amine. Freshly prepared NaOMe in anhydrous MeOH was added (1.5 eq., 100 mL) *via* cannula followed by the addition of FAME (1.2 eq.). A condenser was attached and the mixture was stirred under N_2_ at 55 °C overnight. Subsequently, 60 g of Amberlyst® 15 H+ form was added till pH ∼ 6 in order to protonate the small amount of hydrolyzed FAME, so as to wash it out with heptane. The Amberlite was removed by filtration, MeOH was evaporated and the resulting dark-red mixture was sonicated in warm i-PrOH for around 5 min. The resulting mixture was passed through a pad of silica gel and the silica was washed with iPrOH of 40 °C. (Note: at higher *T*, impurities start to pass the silica gel, in that case the obtained mixture can be recrystallized from hot i-PrOH). iPrOH was evaporated, resulting in a white powder that was washed with heptane to remove and recover remaining FAME and fatty acid.

### Analysis of the surfactants with cryo-electron microscopy

The desired amount of fructose-based surfactant was dissolved in water at a concentration >0.2 wt% and at a temperature above the Krafft temperature. Upon the solution turning clear, the samples were cooled to room temperature and then were kept at 4 °C for at least 24 h. A sample of a few microliters was placed on a Quantifiol 3.5/1 holey carbon-coated grid (Quantifiol GmbH, Jena, Germany) and blotted with filter paper. The grids were vitrified by plunging into liquid ethane and transferred to a Gatan model 626 cryostage. The grids were examined in a Tecnai T20 (FEI, Eindhoven, The Netherlands) cryo-electron microscope operating at 200 keV, under low-dose conditions. Cryo-electron diffraction was carried out at a camera length of 770 mm and exposure times between 5 and 10 s.

## Author contributions

The study was initiated by A.J.M. Synthesis and analysis were performed by H.-C.L., J.P.K., M.K., and I.K., Electron Microscopy was carried out by M.C.A.S., expert input on synthesis and surfactant properties was provided by B.F. The manuscript was written by H.-C.L. and A.J.M. with input from all authors.

## Conflicts of interest

H.-C. L., B. F. and A. J. M. are authors on a patent application led by the University of Groningen that covers the majority of the work described in this paper.

## Supplementary Material

GC-026-D4GC00399C-s001

GC-026-D4GC00399C-s002
