# Supplementary material for: The synthesis of fructose-based surfactants
Source: Green Chem. 2024 Mar 13;26(8):4715–22. doi: 10.1039/d4gc00399c (PMC11033973; doi:10.1039/d4gc00399c)
Supplement: GC-026-D4GC00399C-s001 [file GC-026-D4GC00399C-s001.pdf]

Electronic Supplementary Material (ESI) for Green Chemistry.
This journal is © The Royal Society of Chemistry 2024

## Supporting Information

# The synthesis of fructose-based surfactants

Hung-Chien Lin, Marios Kidonakis, J. P. Kaniraj, Ihor Kholomieiev, Balint Fridrich, Marc C. A. Stuart, Adriaan J. Minnaard*

### Surface Tension Measurements

The surface tension was measured by the pendant drop method with an optical tensiometer. The analysis mode was set to Young-Laplace. The light phase was pentane and the heavy phase was water at 20 °C. The pendant drop of the sample was made in a cuvette filled with pentane and recorded by the camera for 10 s at 10 FPS. The surface tension over 10 s was calculated according to the set parameters and the recorded pendant drop.

**Table S1. Investigation of the fructose-alcohol ratio for an optimum yield of 2**

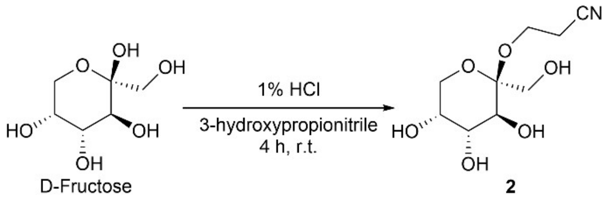


| Entry | Fructose (g) | Fructose/Alcohol ratio (g/mL) | Stirring Method | Yield |
|---|---|---|---|---|
| 1 | 5 g | 0.125 | Magnetic | N/A |
| 2 | 5 g | 0.25 | Magnetic | 38% |
| 3 | 5 g | 0.5 | Magnetic | 68% |
| 4 | 5 g | 1 | Magnetic | 61% |
| 5 | 10 g | 0.5 | Overhead stirring | 43% |
| 6 | 10 g | 1 | Overhead stirring | 54% |

1% HCl was made in situ by adding acetyl chloride to 3-hydroxypropionitrile (volume ratio = 1:40)

**Table S2. The effect of acid concentration on yield and precipitation**

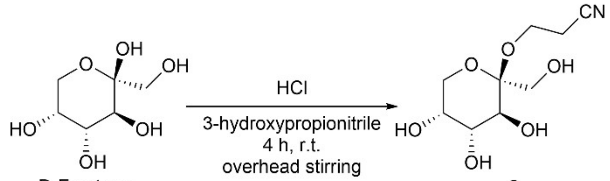


D-Fructose
fructose-alcohol
ratio = 1 g/mL

**2**

| Entry | AcCl : alcohol | Yield | Remark |
|---|---|---|---|
| 1 | 1:40 (= 1% HCl) | 54% | Thick slurry formed in a few min |
| 2 | 1:100 | 66% | Thick slurry formed in 30 min |
| 3 | 1:200 | 59% | Thick slurry formed in 30 min |
| 4 | 1:430 | 61% | Thick slurry formed in 2 h |

The reaction of entry 1 was performed with 10 g of fructose
The reaction of entry 2-4 were performed with 30 g of fructose

**Table S3. Physical characteristics of the fructo-surfactants (CMC and surface tension at 25 °C) and the reported values of available glucose-based surfactants.**

| | **CMC (mM)** | **Surface tension (mN/m)** | **Tm (°C)** | **Tk (°C)** |
|---|---|---|---|---|
| **8** (**$C_8$**) | 61 | 7.7 (35.0) | 101 | n.d. |
| **9** (**$C_{10}$**) | 6.4 | 4.7 | 81 | 36 |
| **10** (**$C_{12}$**) | 0.6 | 2.4 | 104 | 53 |
| **11** (**$C_{16}$**) | n.d. | n.d. | 110 | 75 |
| **12** (unsaturated **$C_{18}$**) | n.d. | n.d. | 107 | n.d. |
| **$C_8$**-α-Glc[1] | 9.4 | - | - | 40 |
| **$C_8$**-β-Glc[1,2] | 21 | (30.1) | - | <0 |
| **$C_{10}$**-β-Glc[2] | 2.2 | (29.0) | - | 26 |
| **$C_8$**-MEGA[3–5] | 69.0 | (35.5, at 30 °C) | - | ~0 |
| **$C_{10}$**-MEGA [3–5] | 6.7 | (32.0, at 30 °C) | - | 31 |

Glc = glucopyranoside, MEGA = N-methyl glucamide. The surface tension was determined in a water-pentane system. The values in bracket are those of a water-air system. Tm = melting point of the pure solid (in practice a trajectory of 2-3 degrees), Tk = phase transition temperature of surfactant at 0.2 wt% in water (approximate Krafft point)

**Procedure for the Nile Red CMC determination assay**[6]

A stock solution of Nile-Red in DCM (0.5 mM, 10 mL) was prepared. 3 [illegible]L of the stock solution was placed in a 4 mL vial. The vial was placed under vacuum until complete evaporation of DCM. An aqueous stock solution of the C10 surfactant was prepared in demi-water (100.13 mM, 10 mL) and an aliquot was added to the vial containing the Nile Red, which was then diluted to the desired concentration (final volume 3 mL).

Nile Red fluorescence was measured on an FS5 Spectrofluorometer (Edinburgh Instruments spectrometer) at 25 ºC, in an SC-05 Standard Cuvette Holder, and using type 23/Q/10 cuvettes (quartz, path length: 10mm). Fluorescent emission was measured from 570 to 750 nm. The equivalence point reflects the CMC.

| C (mM) | λmax |
|---|---|
| 0.1 | 667.2 |
| 1 | 663 |
| 2 | 657.3 |
| 4 | 650.3 |
| 5 | 641.8 |
| 5.5 | 639.9 |
| 6 | 620 |
| 6.5 | 611 |
| 7 | 609.5 |

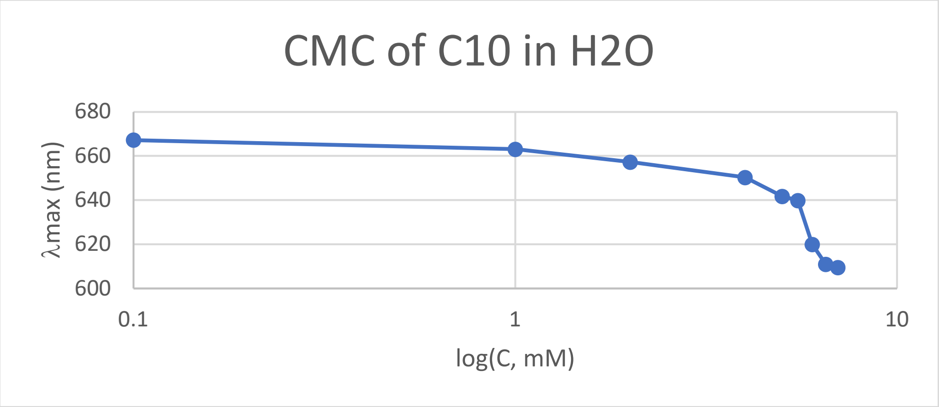


Graph S1 CMC-determination of the C10 surfactant using a Nile Red assay

## Foaming measurements

**General foaming test**[7]

300 mg of the C10 surfactant **9** was pulverized and dissolved in 30 mL distilled water.
The solution was transferred to a 100 mL volumetric cylinder and stirred for 3 min (overhead stirrer, Heidolph TR 20 Radial Flow Impeller, 2000 rpm). The foam volume (mL) was monitored after 3, 5 and 10 min. The procedure was repeated for the commercial surfactant sodium dodecyl sulfate (SDS).

**Table S4**

| | Foam (mL) after 3 min stirring | Foam volume after 3 min | Foam volume after 5 min | Foam volume after 10 min |
|---|---|---|---|---|
| C10 surfactant **9** | 36 mL | 36 mL | 36 mL | 34 mL |
| SDS | 36 mL | 36 mL | 36 mL | 36 mL |

**Speed of foam formation**

A 1 wt% surfactant (SDS, $C_8$ or $C_{10}$ (**9**) surfactant) solution was slowly poured into a 100 ml beaker, taking care to prevent foam formation. The height of the solution in the beaker was recorded. The solution was agitated with a Fisher Scientific Power Gen 1000 homogenizer for 5 s, after which the homogenizer was removed and the height of the foam was measured. After the measurement the agitation was continued and measurements were taken similarly at 10, 15, 20, 25 and 60 s. The obtained results are presented in Table S5.

**Table S5, Speed of foam formation of SDS, $C_8$ and $C_{10}$ surfactants**

| | Initial solution height - 16 mm | | Initial solution height - 20 mm | | Initial solution height - 18 mm |
|---|---|---|---|---|---|
| Time (s, SDS) | Foam height (mm, SDS) | Time (s, C10) | Foam height (mm, C10) | Time (s, C8) | Foam height (mm, C8) |
| 0 | 0 | 0 | 0 | 0 | 0 |
| 5 | 27 | 5 | 31 | 5 | 20 |
| 10 | 28 | 10 | 35 | 10 | 27 |
| 15 | 30 | 15 | 35 | 15 | 28 |
| 20 | 30 | 20 | 35 | 20 | 30 |
| 25 | 30 | 25 | 35 | 25 | 31 |
| 60 | 33 | 60 | 36 | 60 | 31 |

**Foam stability**

A 1 wt% surfactant (SDS, $C_8$ or $C_{10}$ (**9**)) solution was slowly poured into a 100 ml beaker, taking care to prevent foam formation. The height of the solution in the beaker was recorded. The solution was agitated with a Fisher Scientific Power Gen 1000 homogenizer for 60 s, after which the homogenizer was removed and the height of the foam was measured. The solution was left to stand and the height of foam was measured periodically in the next 60 min. The obtained results are presented in Table S6.

**Table S6. Foam stability of SDS, $C_8$ and $C_{10}$ (9)surfactants**

| | Initial solution height - 25 mm | | Initial solution height - 25 mm | | Initial solution height - 18 mm |
|---|---|---|---|---|---|
| Time (min, SDS) | Foam height (mm, SDS) | Time (min, C10) | Foam height (mm, C10) | Time (min, C8) | Foam height (mm, C8) |
| 0 | 38 | 0 | 40 | 0 | 31 |
| 4 | 29 | 7 | 26 | 2 | 25 |
| 12 | 23 | 14 | 25 | 6 | 23 |
| 19 | 18 | 20 | 23 | 9 | 21 |
| 27 | 17 | 26 | 22 | 14 | 21 |
| 32 | 15 | 34 | 21 | 18 | 18 |
| 38 | 14 | 39 | 20 | 23 | 17 |
| 46 | 11 | 44 | 20 | 28 | 17 |
| 52 | 9 | 52 | 18 | 34 | 16 |
| 64 | 7 | 61 | 17 | 39 | 16 |
| | | | | 44 | 16 |
| | | | | 48 | 15 |
| | | | | 51 | 15 |

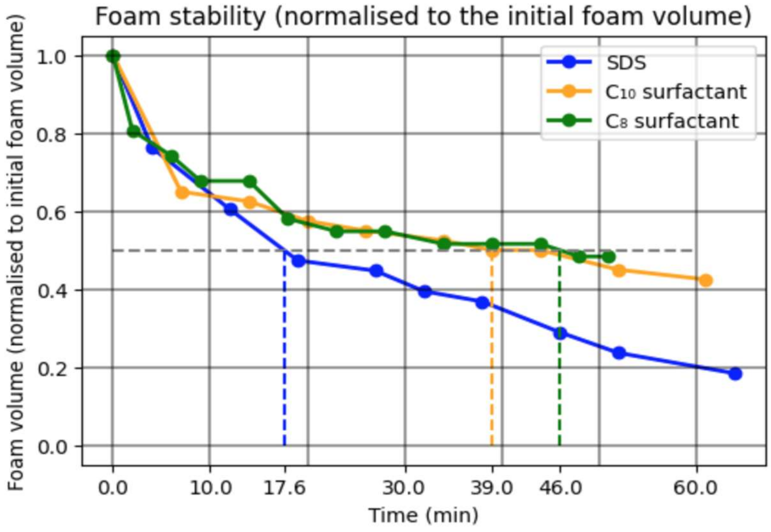


Graph S2 Foam stability normalized to the initial foam volume

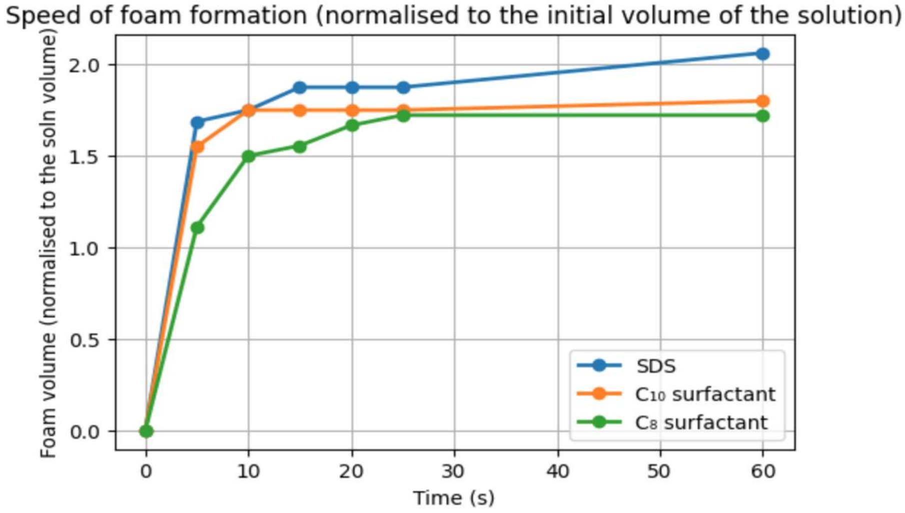


Graph S3 Speed of foam formation normalized to the initial solution volume

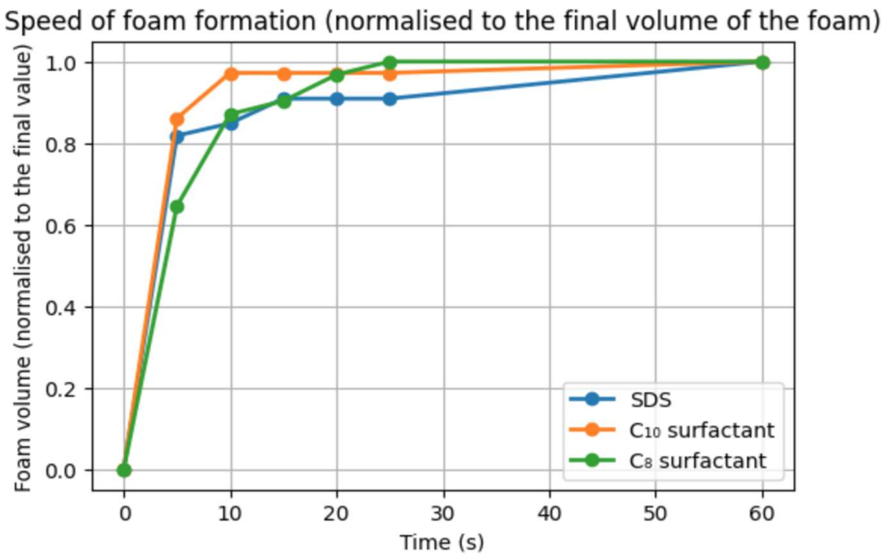


Graph S4 Speed of foam formation normalized to the final volume of the foam

**Emulsification measurements**

25 v% of sunflower oil and 75 v% of a 1% surfactant solution were placed in a beaker and the mixture was agitated with a Fisher Scientific Power Gen 1000 homogenizer for 60 s, after which the homogenizer was removed. The obtained emulsions were left to separate.

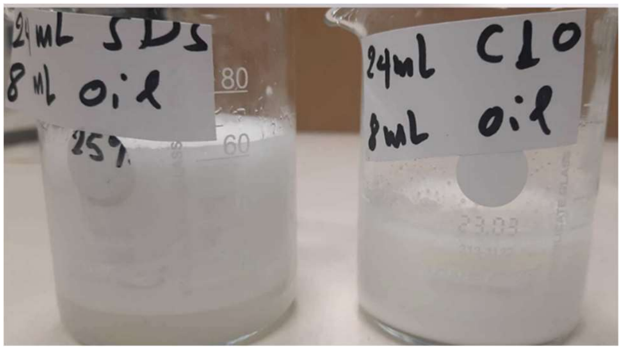


Fig S1 Comparison of the emulsion stability of SDS (left) and C10 fructose surfactant **9** (right).

**References**


1 T. Gaudin, H. Lu, G. Fayet, A. Berthauld-Drelich, P. Rotureau, G. Pourceau, A. Wadouachi, E. Van Hecke, A. Nesterenko and I. Pezron, *Adv. Colloid Interface Sci.*, 2019, **270**, 87–100.

2 K. Shinoda, T. Yamaguchi and R. Hori, *Bull. Chem. Soc. Jpn.*, 1961, **34**, 237–241.

3 M. Okawauchi, M. Hagio, Y. Ikawa, G. Sugihara, Y. Murata and M. Tanaka, *Bull. Chem. Soc. Jpn.*, 1987, **60**, 2719–2725.

4 J.-S. Ko, S.-W. Oh, Y.-S. Kim, N. Nakashima, S. Nagadome and G. Sugihara, *J. Oleo Sci.*, 2004, **53**, 109–126.

5 M. Alexeeva, T. Churjusova, N. Smirnova and A. Vlasov, *Fluid Phase Equilib.*, 1997, **136**, 173–183.

6 M. C. A. Stuart, J. C. van de Pas, J. B. F. N. Engberts, *J. Phys. Org. Chem.* 2005, **18**, 929–934.

7 L. M. Jansen, K. W. M. van Rijbroek, P. C. den Bakker, D. J. Klaassen-Heshof, W. J. B. Kolkman, N. Venbrux, V. Migchielsen, J. Hutzezon, W. B. Lenferink, S. Lucker, A. Ranoux, H. W. C. Raaijmakers, T. J. Boltje, *ACS Sus. Chem. & Eng.* 2023, **11**, 16117 – 16123.

**Preparation of cyanoethyl-β-fructopyranoside 2**

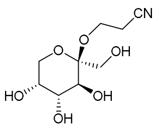

**2**

**From fructose**

250 ml of 3-hydroxypropionitrile and 6.25 ml of acetyl chloride were added to a 5 L round-bottom flask and mixed using an overhead stirrer for 15 min. 250 g of D-fructose was added to the flask and stirring was continued for 4 h. Part of the fructose gradually dissolved during the first 30 min, after which precipitation started and the mixture became progressively more viscous. Subsequently, 2 L of isopropanol was added and stirring was continued for 45 min in an ultrasonic bath. The obtained slurry was filtered (450 mbar, porosity 3 glass filter) and the cake was washed with isopropanol until no 3-hydroxypropionitrile was detected with $^{1}$H NMR. Drying *in vacuo* yielded 189 g (59%) of **2** as a white solid. **$^{1}$H NMR** (400 MHz, DMSO-$d_6$) δ 4.72-4.34 (m, 4H), 3.75-3.66 (m, 3H), 3.65-3.55 (m, 3H), 3.54-3.48 (m, 3H), 2.78-2.69 (m, 2H) **$^{13}$C NMR** (101 MHz, DMSO-$d_6$) δ 119.5, 100.6, 69.1, 69.0, 68.9, 64.1, 62.4, 56.0, 18.4 **m.p.** = 120 $^{o}$C **[α]** = -130.6$^{o}$ **HRMS** (ESI) calculated for $C_8H_{15}ClO_6$ ($[M+Na]^+$): 256.0792, found: 256.0788.

*Synthesis of **2** from inulin*

In a 250 mL round bottom flask, 0.5 mL of acetyl chloride was added to 20 mL of 3-hydroxypropionitrile. The solution was stirred for 15 min at rt with an overhead stirrer. 10 g of inulin was added to the solution and left stirring at rt for 4 d. Subsequently, the resulting slurry was mixed with isopropanol to give a suspension. The suspended solid was collected by filtration with reduced pressure (using a fritted funnel with porosity 2 and 800 mbar). The collected solid was washed with isopropanol and dried under vacuum. 46% yield of **2** was obtained as a white solid.

*Synthesis of **2** from sucrose*

In a 250 mL round bottom flask, 2.5 mL of acetyl chloride was added to 100 mL 3-hydroxypropionitrile. The solution was stirred for 15 min at r.t. with an overhead stirrer. 25 g of sucrose was added to the solution and left stirring at rt for 4 d. The remaining solid (undissolved sucrose) was collected by filtration and the filtrate was left stirring at r.t. for another 2 d. A small amount of precipitate was observed. The solid was collected by filtration to give a mixture of D-glucose and fructoside **2** in a 24% yield (combined yield).

**Preparation of aminoethyl-β-fructopyranoside 3**

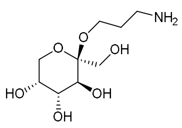

**3**

**By hydrogenation with Raney Nickel.**

To a 1 L three-necked flask, 8 gr of an aqueous Raney-Nickel suspension was added under nitrogen atmosphere. In order to remove the water, 20 mL of MeOH was added, the slurry was stirred for a few s, stirring was halted and the MeOH was removed with a syringe. This procedure was repeated twice. At that point, 100 mL of MeOH was added, the system was evacuated and backfilled with nitrogen 3 times. A $H_2$ balloon was attached, the RaNi/MeOH suspension was flushed with hydrogen for around 5 to 10 min, and 20 g of **2**

was added together with 100 mL of MeOH. Hydrogen was led through the solution for an additional 10 min. Subsequently, 5 eq of $NH_3$ in MeOH (7 M) was added and the system was heated to 45 °C overnight. Upon completion, stirring was terminated whereupon the RaNi stuck on the magnetic stirring bar. The mixture was passed through a pad of celite, and the celite was washed with MeOH. The material was collected in a one-neck flask. Note: the amine **3** is hydroscopic and should be stored under inert atmosphere. **$^1$H NMR** (400 MHz, $CD_3OD$) δ 3.91 (d, *J* = 9.8 Hz, 1H), 3.88 – 3.82 (m, 1H), 3.81 – 3.75 (m, 2H), 3.75 – 3.71 (m, 2H), 3.71 – 3.67 (m, 1H), 3.61 (dt, *J* = 6.1, 3.1 Hz, 2H), 2.76 (d, *J* = 6.6 Hz, 2H), 1.74 (t, *J* = 6.5 Hz, 2H) **$^{13}$C NMR** (101 MHz, $CD_3OD$) δ 101.2, 71.5, 71.0, 70.9, 65.3, 63.6, 60.2, 40.1, 33.6 **HRMS** (ESI) calculated for $C_9H_{19}NO_6$ ([M+H]$^+$): 238.1285, found: 238.1282.

**Preparation of $C_8$-fructose-based surfactant 8**

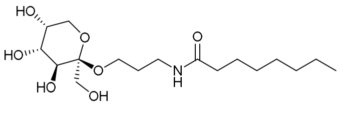


**8**

Compound **8** was synthesized according to described NaOMe-mediated amidation to give 60% yield of the off-white solid product. The resulting product was virtually pure but was further purified by column chromatography (10% MeOH in DCM) if high purity was needed. **$^1$H NMR** (400 MHz, $CD_3OD$) δ 3.91 (d, *J* = 9.8 Hz, 1H), 3.86 – 3.83 (m, 1H), 3.81 – 3.77 (m, 1H), 3.77 – 3.74 (m, 1H), 3.74 – 3.69 (m, 2H), 3.66 (dd, *J* = 12.3, 1.9 Hz, 1H), 3.62 – 3.51 (m, 2H), 3.31 – 3.25 (m, 2H), 2.18 (t, *J* = 7.5 Hz, 2H), 1.76 (td, *J* = 6.3, 3.3 Hz, 2H), 1.60 (t, *J* = 7.3 Hz, 2H), 1.39 – 1.26 (m, 8H), 0.94 – 0.86 (m, 3H). **$^{13}$C NMR** (101 MHz, $CD_3OD$) δ 176.3, 101.6, 71.5, 71.0, 70.8, 65.3, 63.6, 59.6, 37.8, 37.2, 32.9, 30.6, 30.3, 30.2, 27.1, 23.7, 14.4 **m.p.** = 101 °C **[α]** = -86.0° **HRMS** (ESI) calculated for $C_{17}H_{33}NO_7$ ([M+H]$^+$): 364.2330, found: 364.2330.

**Preparation of $C_{10}$-fructose-based surfactant 9**

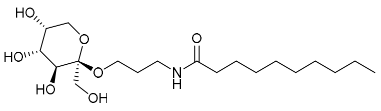


**9**

Compound **9** was synthesized according to described NaOMe-mediated amidation to give 77% yield of the off-white solid product. The resulting product was virtually pure but was purified by column chromatography (10% MeOH in DCM) if high purity was needed. **$^1$H NMR** (400 MHz, $CD_3OD$) δ 3.91 (d, *J* = 9.8 Hz, 1H), 3.87 – 3.83 (m, 1H), 3.79 (dd, *J* = 9.8, 3.5 Hz, 1H), 3.77 – 3.74 (m, 1H), 3.74 – 3.69 (m, 2H), 3.68 – 3.62 (m, 1H), 3.62 – 3.50 (m, 2H), 3.31 – 3.25 (m, 2H), 2.18 (t, *J* = 7.6 Hz, 2H), 1.76 (td, *J* = 6.5, 3.4 Hz, 2H), 1.60 (t, *J* = 7.2 Hz, 2H), 1.40 – 1.20 (m, 12H), 0.90 (t, *J* = 6.6 Hz, 3H). **$^{13}$C NMR** (101 MHz, $CD_3OD$) δ 176.3, 101.6, 71.5, 71.0, 70.8, 65.3, 63.5, 59.6, 37.8, 37.2, 33.0, 30.61, 30.57, 30.5, 30.4, 30.3, 27.1, 23.7, 14.4 **m.p.** = 81 °C **[α]** = -74.8° **HRMS** (ESI) calculated for $C_{19}H_{37}NO_7$ ([M+H]$^+$): 392.2643, found: 392.2644.

**Preparation of $C_{12}$-fructose-based surfactant 10**

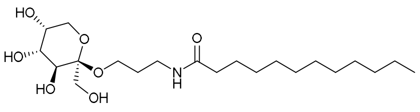

10

Compound **10** was synthesized according to described NaOMe-mediated amidation to give 77% yield of the off-white solid product. The resulting product was virtually pure but was purified by column chromatography (10% MeOH in DCM) if high purity was needed. **$^{1}$H NMR** (400 MHz, $CD_3OD$) δ 3.91 (d, *J* = 9.8 Hz, 1H), 3.87 – 3.83 (m, 1H), 3.79 (dd, *J* = 9.9, 3.4 Hz, 1H), 3.77 – 3.74 (m, 1H), 3.74 – 3.69 (m, 2H), 3.66 (dd, *J* = 12.3, 1.9 Hz, 1H), 3.60 – 3.53 (m, 2H), 3.32 – 3.25 (m, 2H), 2.18 (t, *J* = 7.5 Hz, 2H), 1.76 (td, *J* = 6.4, 3.4 Hz, 2H), 1.60 (t, *J* = 6.9 Hz, 2H), 1.37 – 1.23 (m, 16H), 0.90 (t, *J* = 6.8 Hz, 3H). **$^{13}$C NMR** (101 MHz, $CD_3OD$) δ 176.3, 101.6, 71.5, 71.0, 70.8, 65.3, 63.6, 59.6, 37.8, 37.2, 33.1, 30.73, 30.72, 30.63, 30.57, 30.46, 30.45, 30.3, 27.1, 23.7, 14.4 **m.p.** = 104 $^{o}$C **[α]** = -75.2$^{o}$ **HRMS** (ESI) calculated for $C_{21}H_{41}NO_7$ ($[M+H]^+$):420.2956, found: 420.2954.

**Preparation of $C_{16}$-fructose-based surfactant 11**

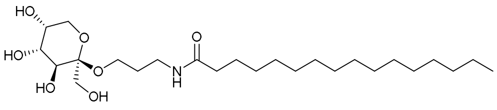

11

Compound **11** was synthesized according to described NaOMe-mediated amidation to give 74% yield of the off-white solid product. The resulting product was virtually pure but was purified by column chromatography (10% MeOH in DCM) if high purity was needed. **$^{1}$H NMR** (400 MHz, DMSO-*d$_6$*) δ 7.66 (t, *J* = 5.5 Hz, 1H), 4.54 – 4.35 (m, 3H), 4.34 – 4.25 (m, 1H), 3.70 (d, *J* = 9.8 Hz, 1H), 3.66 – 3.62 (m, 1H), 3.56 (dd, *J* = 9.9, 3.5 Hz, 1H), 3.54 – 3.43 (m, 4H), 3.38 (td, *J* = 6.3, 3.8 Hz, 2H), 3.08 (q, *J* = 6.5 Hz, 2H), 2.00 (t, *J* = 7.4 Hz, 2H), 1.57 (p, *J* = 6.5 Hz, 2H), 1.44 (p, *J* = 7.0 Hz, 2H), 1.21 (bs, 24H), 0.87 – 0.79 (m, 3H). **$^{13}$C NMR** (101 MHz, DMSO-*d$_6$*) δ 172.0, 100.2, 69.4, 69.1, 69.0, 63.9, 62.0, 57.8, 35.9, 35.4, 31.3, 29.6, 29.03 (three overlapped signals), 29.01 (two overlapped signals), 28.98, 28.9, 28.8, 28.68, 28.66, 25.3, 22.1, 13.9 **m.p.** = 110 $^{o}$C **HRMS** (ESI) calculated for $C_{25}H_{49}NO_7$ ($[M+H]^+$):476.3579, found: 476.3581.

**Preparation of $C_{18}$-fructose-based surfactant 12**

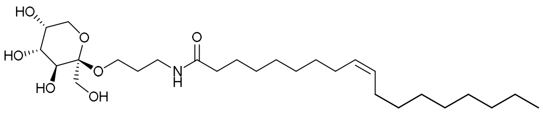

12

Compound **12** was synthesized according to described NaOMe-mediated amidation to give 56% yield of the off-white solid product. The resulting product was virtually pure but was purified by column chromatography (10% MeOH in DCM) if high purity was needed. **$^{1}$H NMR** (400 MHz, $CD_3OD$) δ 5.34 (td, *J* = 4.4, 2.2 Hz, 2H), 3.91 (dd, *J* = 9.8, 1.0 Hz, 1H), 3.86 – 3.83 (m, 1H), 3.79 (ddd, *J* = 9.9, 3.5, 1.0 Hz, 1H), 3.76 – 3.74 (m, 1H), 3.74 – 3.68 (m, 2H), 3.68 – 3.63 (m, 1H), 3.57 (td, *J* = 6.0, 4.2 Hz, 2H), 3.29 (t, *J* = 5.6 Hz, 2H), 2.18 (t, *J* = 7.5 Hz, 2H), 2.03 (q, *J* = 6.1 Hz, 4H), 1.76 (td, *J* = 6.5, 3.5 Hz, 2H), 1.60 (p, *J* = 7.2 Hz, 2H), 1.33 (bs, 11H), 1.30 (bs, 9H), 0.94 – 0.86 (m, 3H). **$^{13}$C NMR** (101 MHz, $CD_3OD$) δ 176.3, 130.9, 130.8, 101.6, 71.5, 71.0, 70.8, 65.3, 63.6, 59.6, 37.8, 37.2, 33.1, 30.8 (two overlapped signals), 30.61, 30.58, 30.44, 30.37, 30.34, 30.32, 30.25, 28.14, 28.13, 27.1, 23.7, 14.5 **m.p.** = 107 $^{o}$C **[α]** = -66.4$^{o}$ **HRMS** (ESI) calculated for $C_{27}H_{51}NO_7$ ($[M+H]^+$):502.3738, found: 502.3733.

**NMR spectra**

cyanoethyl-β-fructopyranoside **2** in DMSO-$d_6$

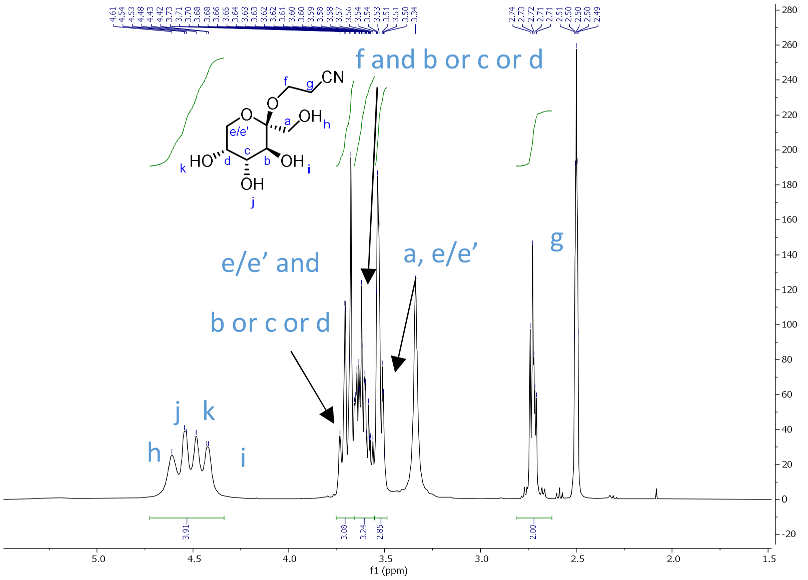


**Figure S2, $^{1}$H-NMR of 2**

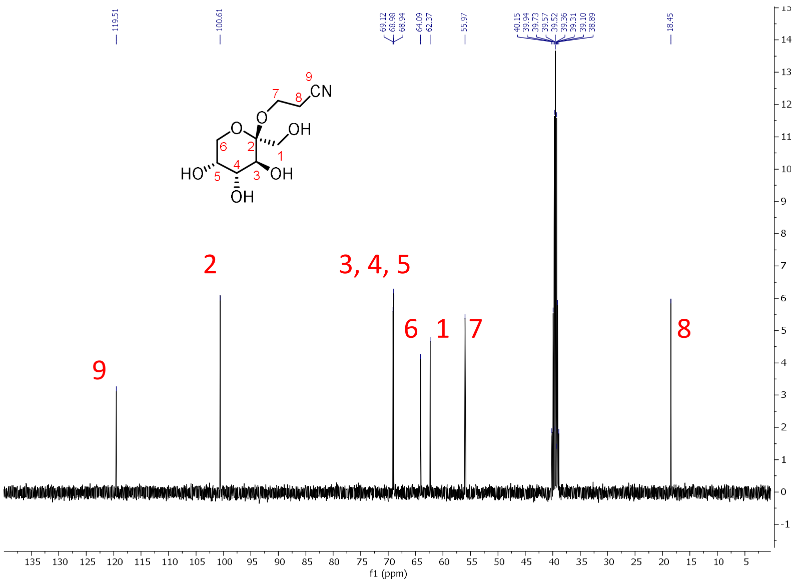


**Figure S3, $^{13}$C-NMR of 2**

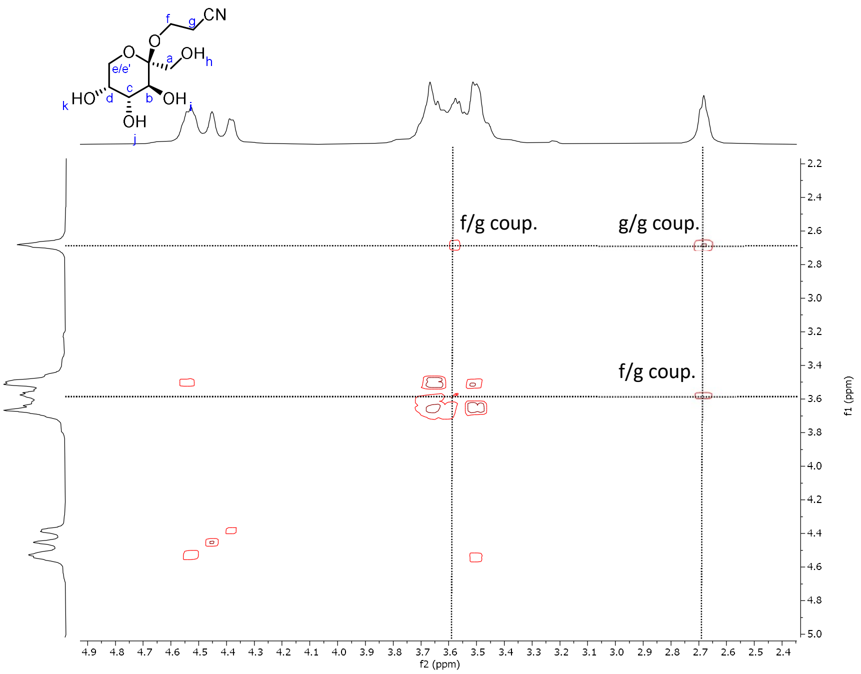


**Figure S4, COSY-NMR of 2**

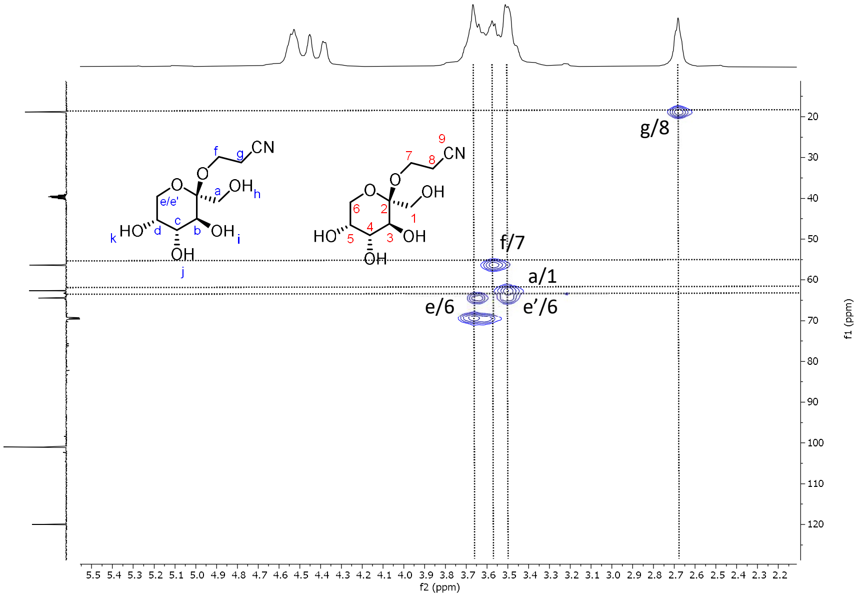


**Figure S5, HSQC-NMR of 2**

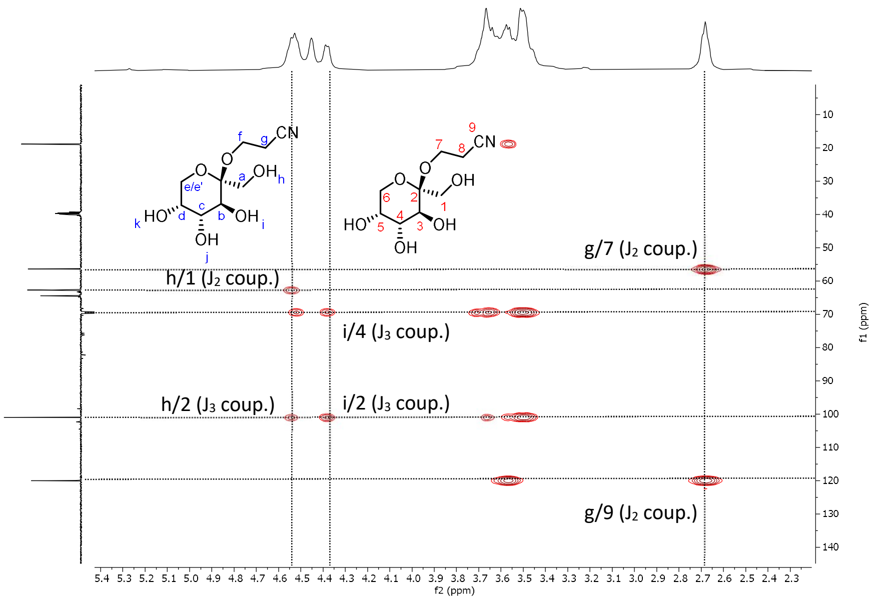


**Figure S6, HMBC-NMR of 2**

aminoethyl-β-fructopyranoside **3** in $CD_3OD$

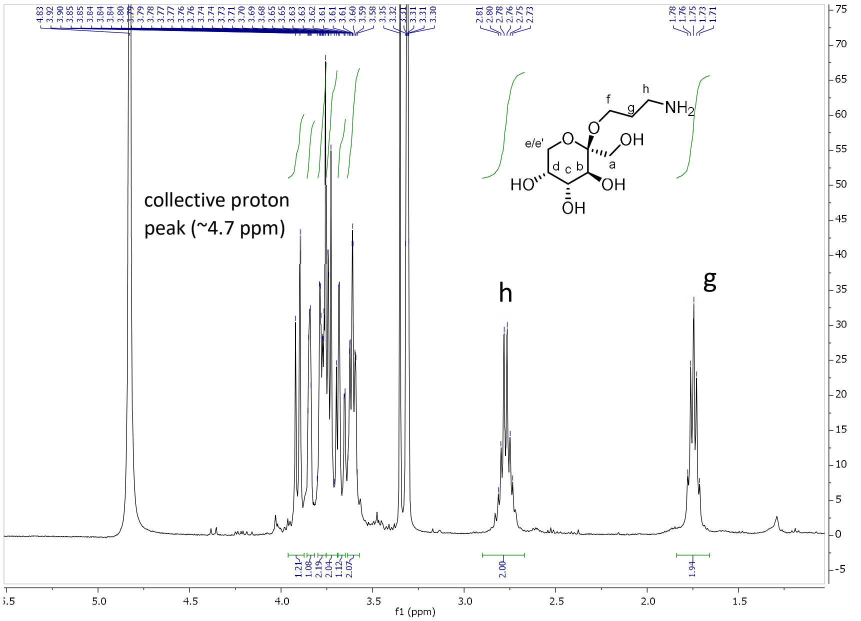


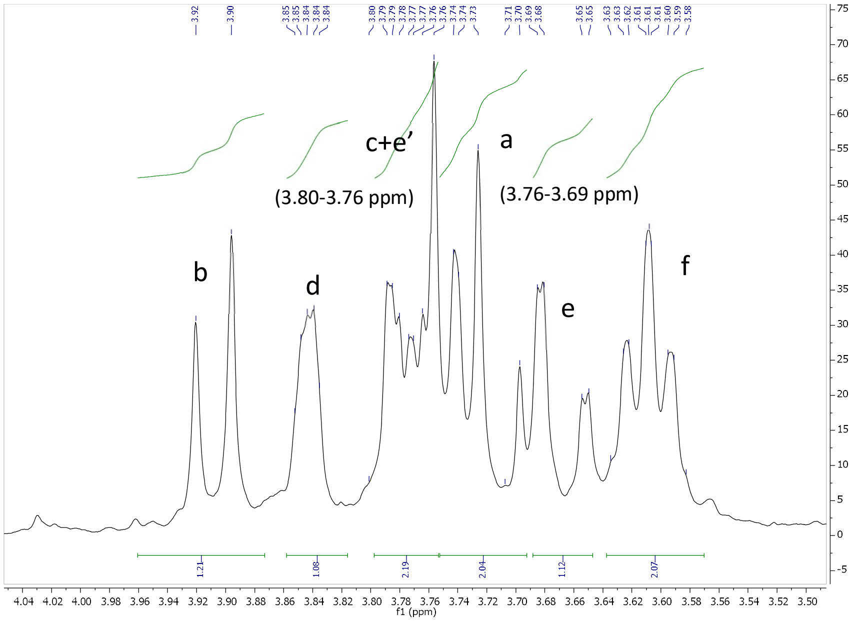


**Figure S7. $^1$H-NMR of 3 in $CD_3OD$**

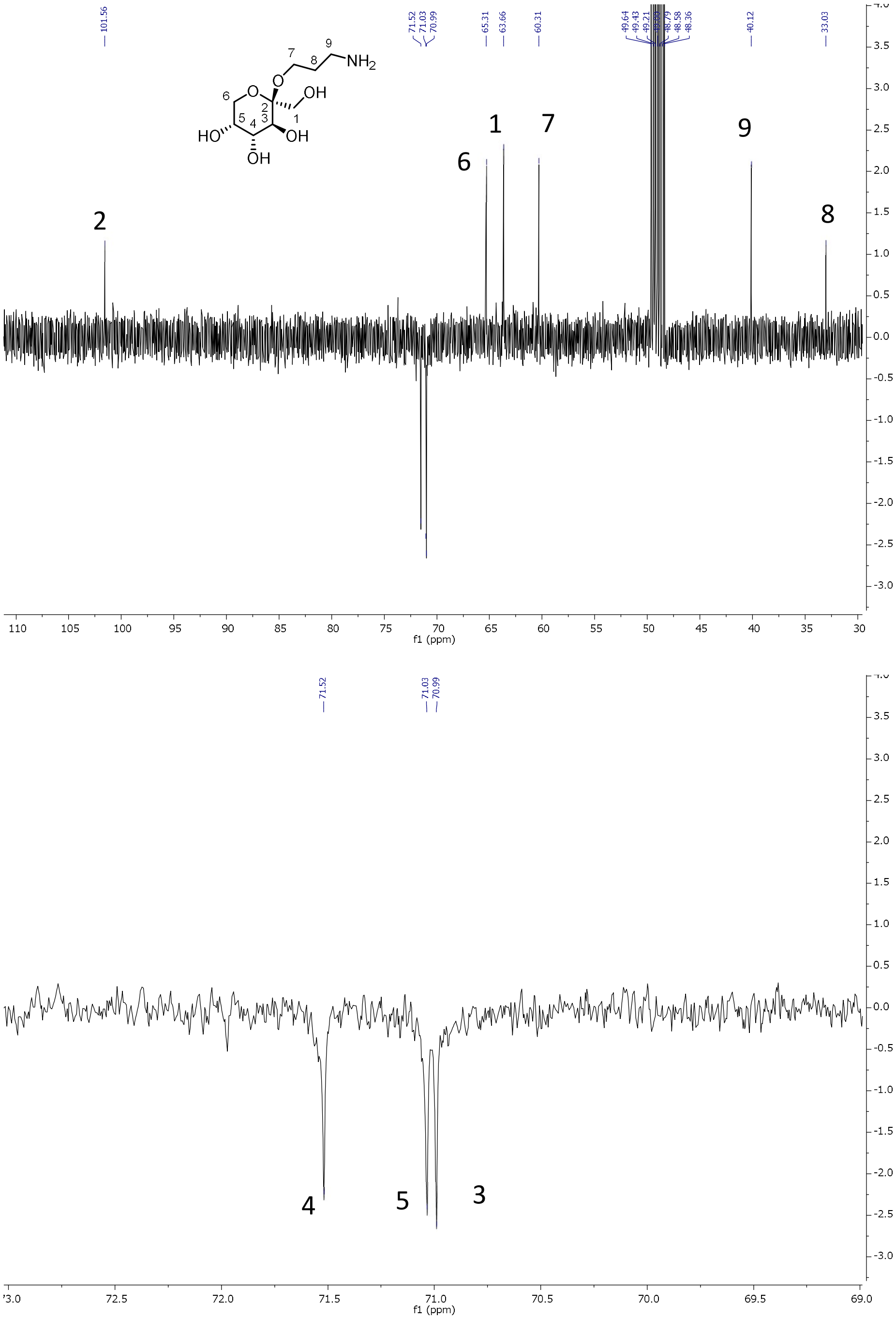


**Figure S8. $^{13}$C-NMR (APT) of 3 in $CD_3OD$**

**$C_8$-fructose-based surfactant 8** in $CD_3OD$

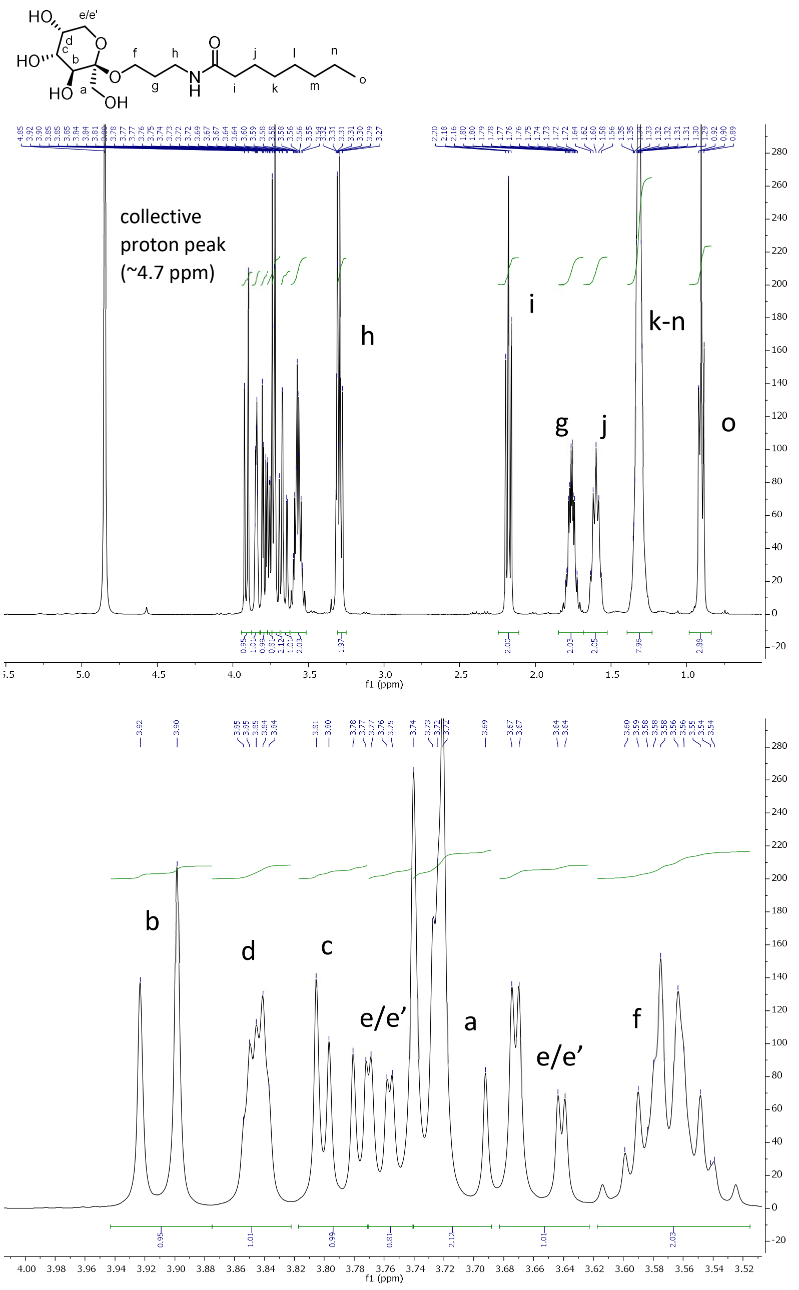


**Figure S9. ¹H-NMR of 8 in $CD_3OD$**

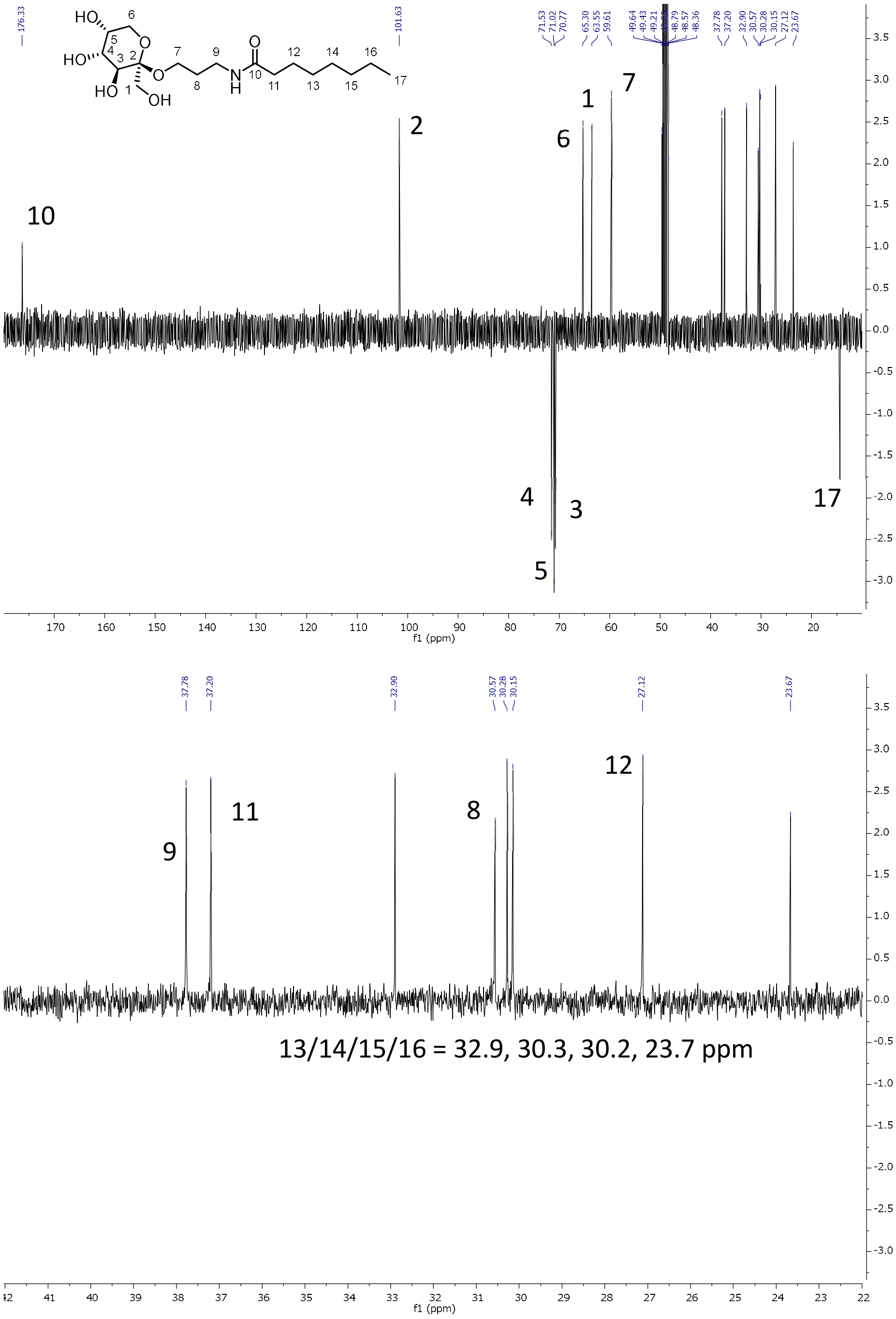


**Figure S10. $^{13}$C-NMR (APT) of 8 in $CD_3OD$**

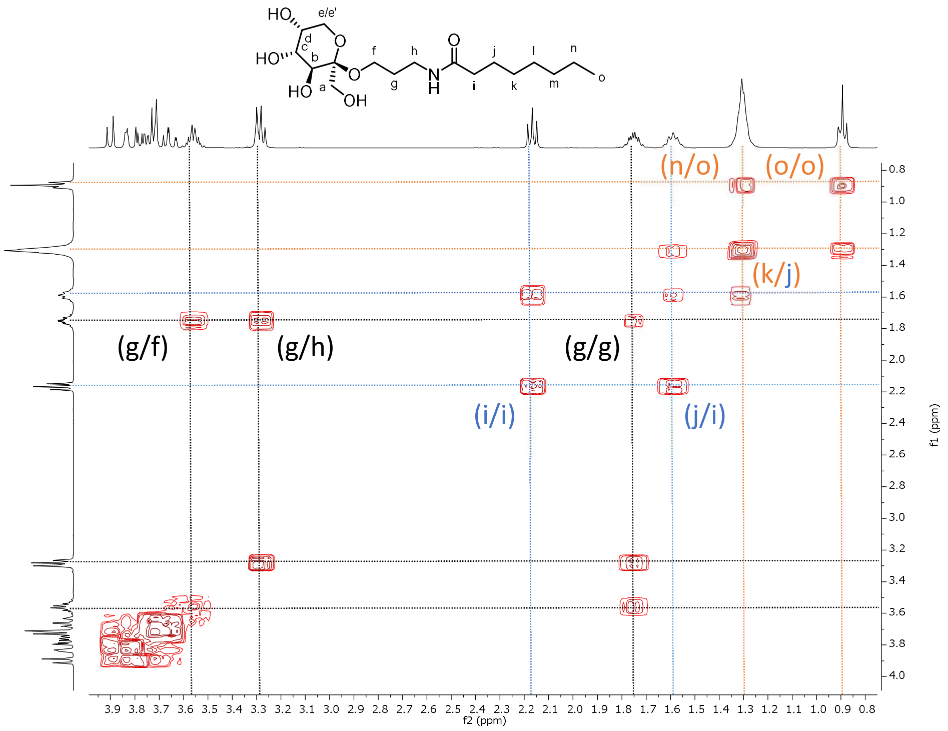


**Figure S11. COSY of 8 in $CD_3OD$**

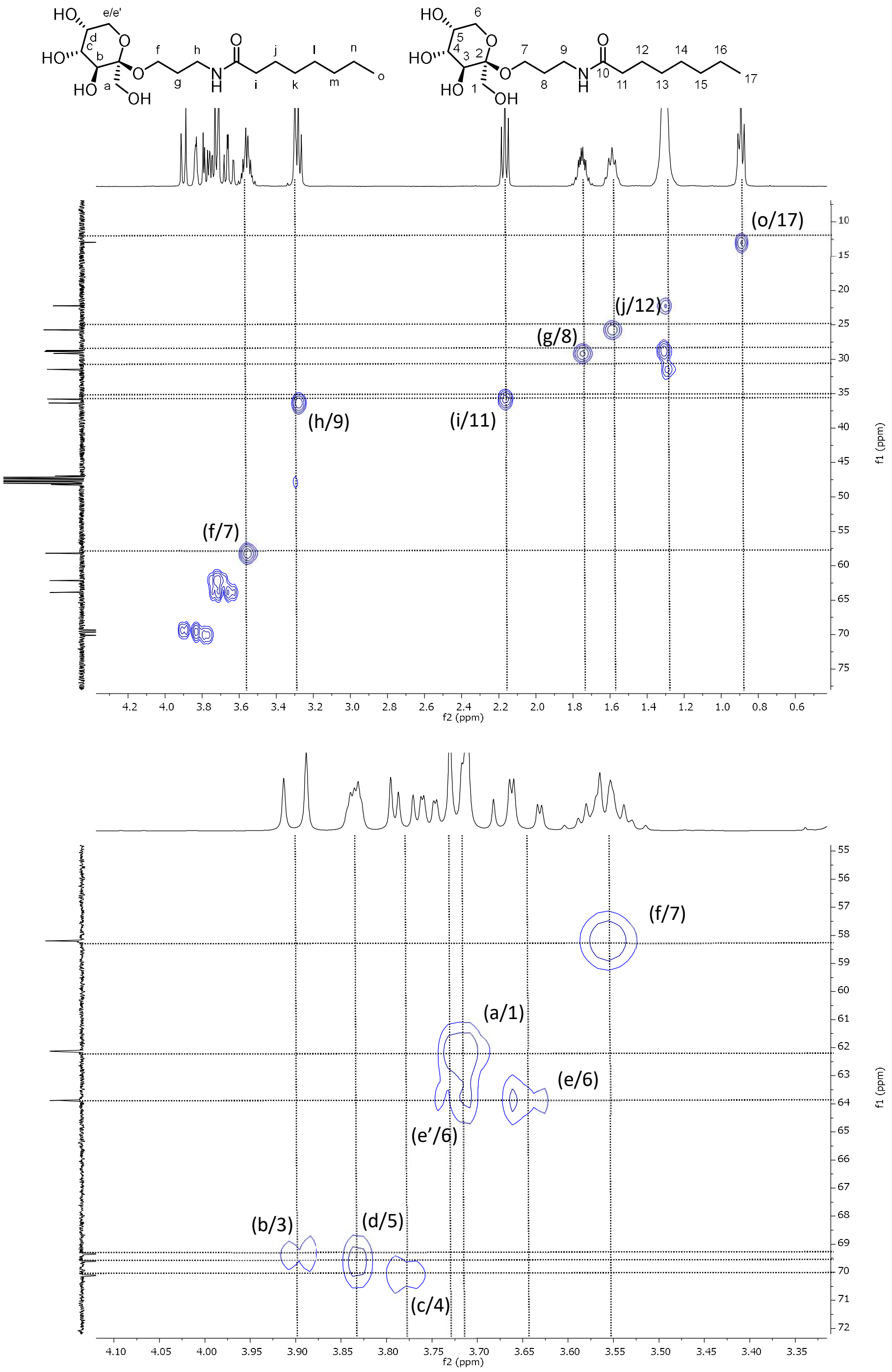


**Figure S12. HSQC of 8 in $CD_3OD$**

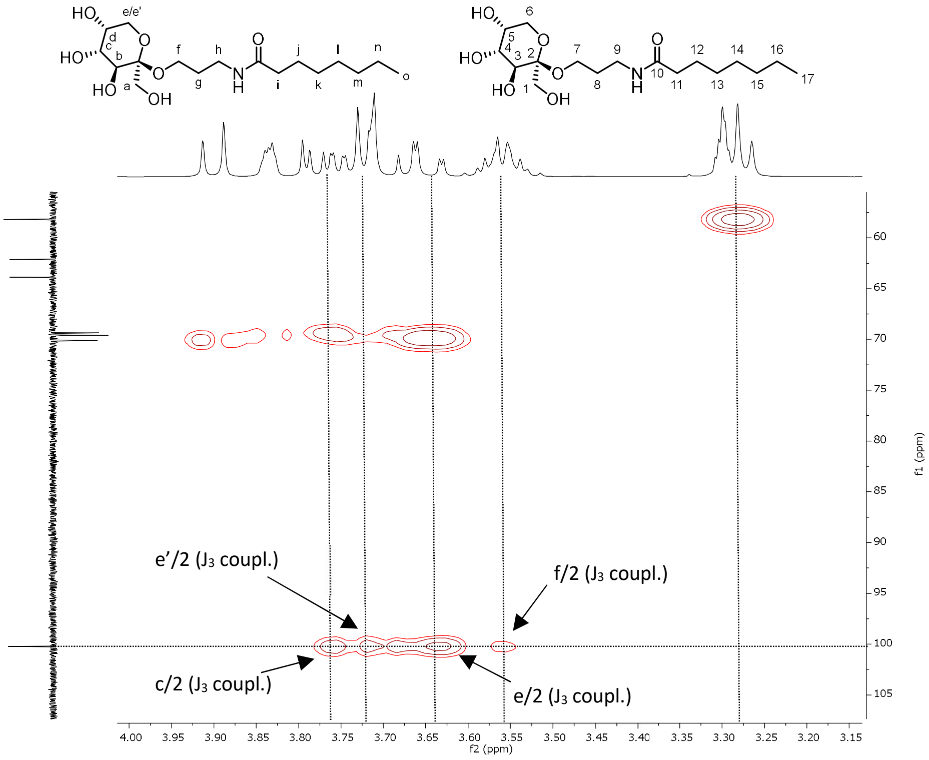


**Figure S13. HMBC of 8 in $CD_3OD$**

**$C_{10}$-fructose-based surfactant 9** in $CD_3OD$

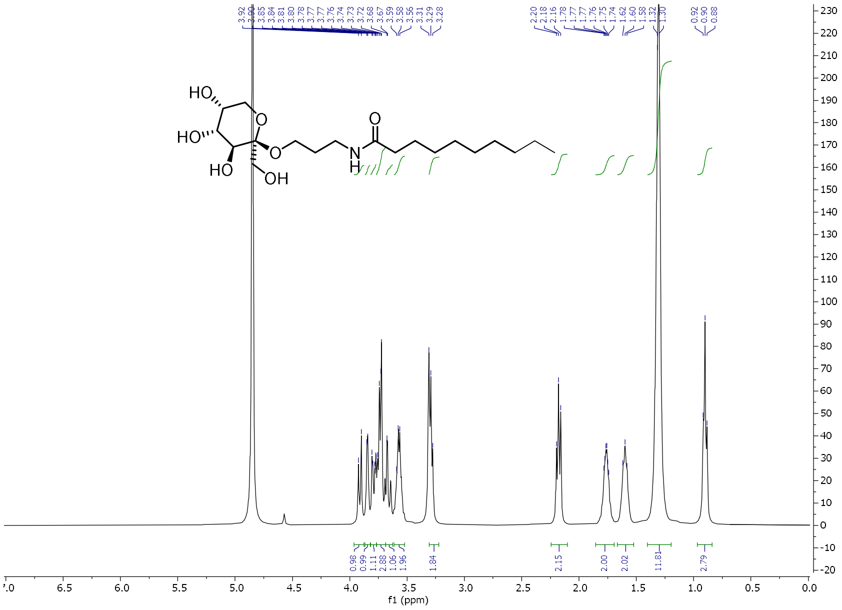


**Figure S14. $^{1}$H-NMR of 9**

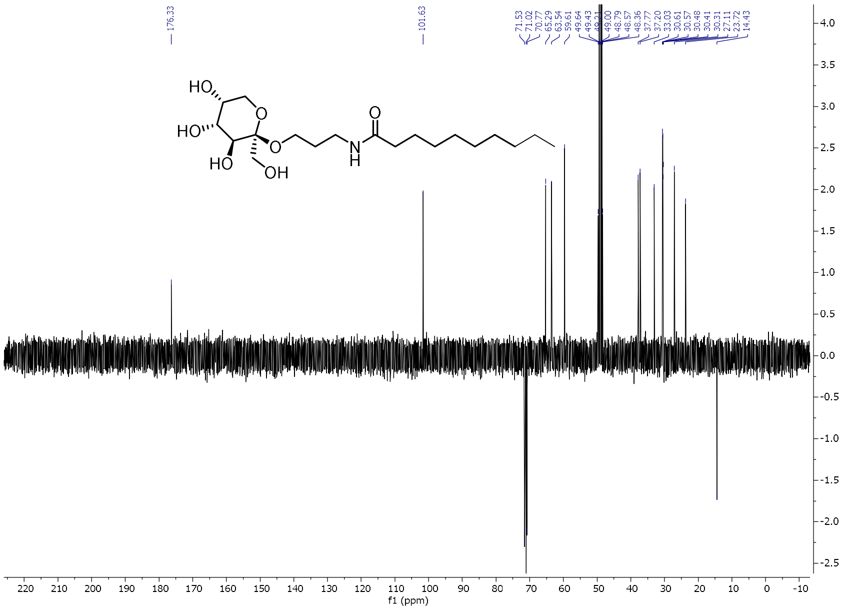


**Figure S15. $^{13}$C-NMR (APT) of 9**

**$C_{12}$-fructose-based surfactant 10** in $CD_3OD$

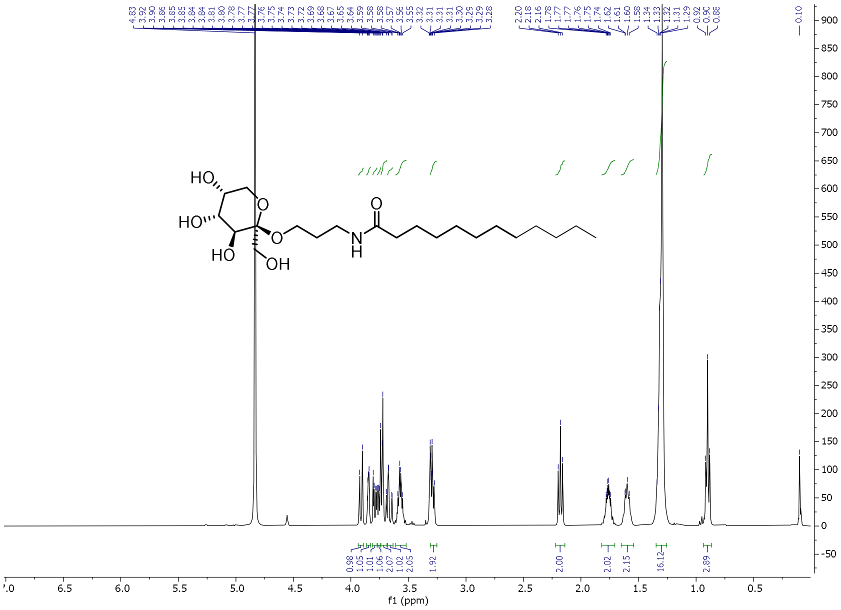

**Figure S16. $^1$H-NMR of 10**

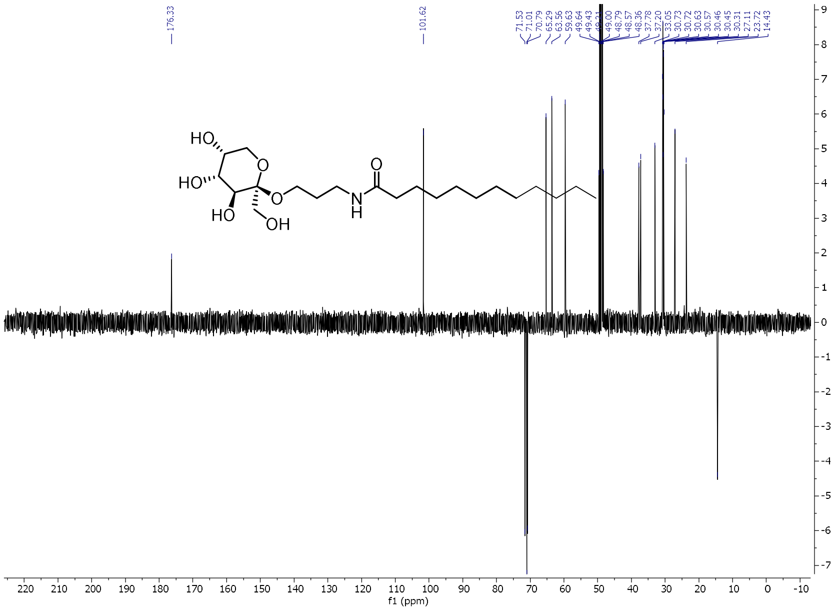

**Figure S17. $^{13}$C-NMR (APT) of 10**

**$C_{16}$-fructose-based surfactant 11** in $CD_3OD$

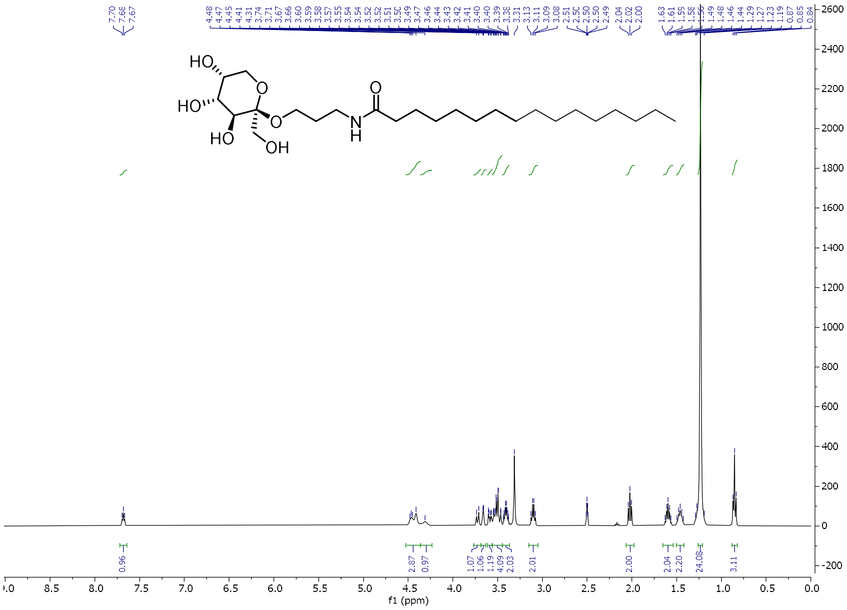

**Figure S18. $^1$H-NMR of 11**

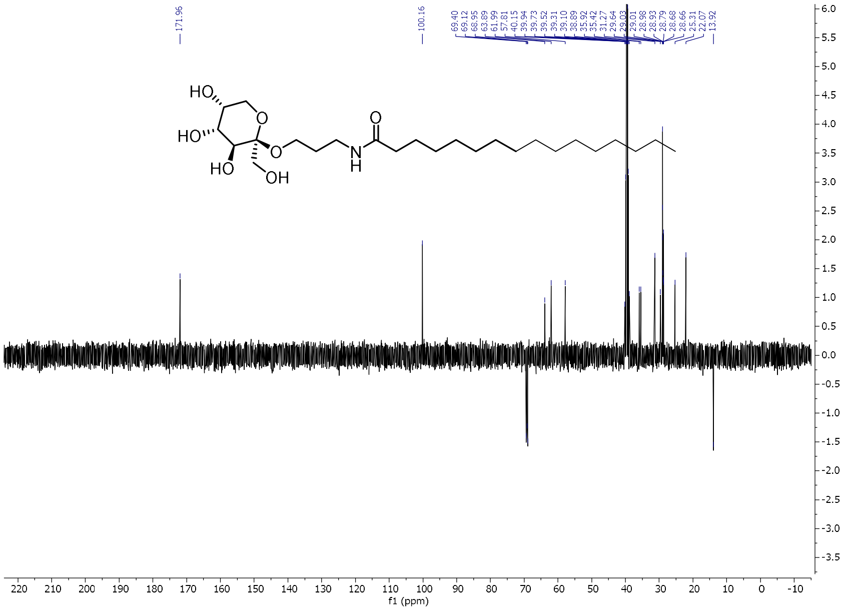

**Figure S19. $^{13}$C-NMR (APT) of 11**

**$C_{18}$-fructose-based surfactant 12** in $CD_3OD$

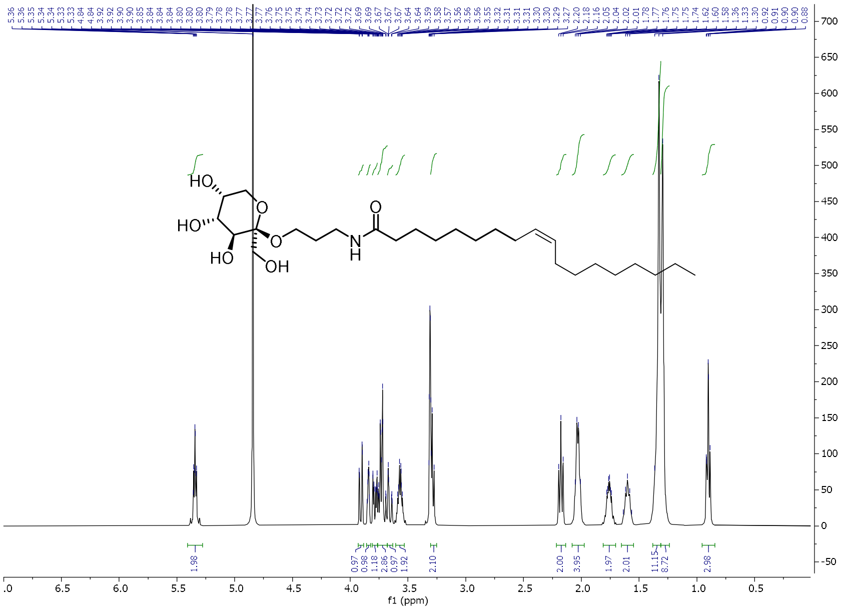

**Figure S20. $^1$H-NMR of 12**

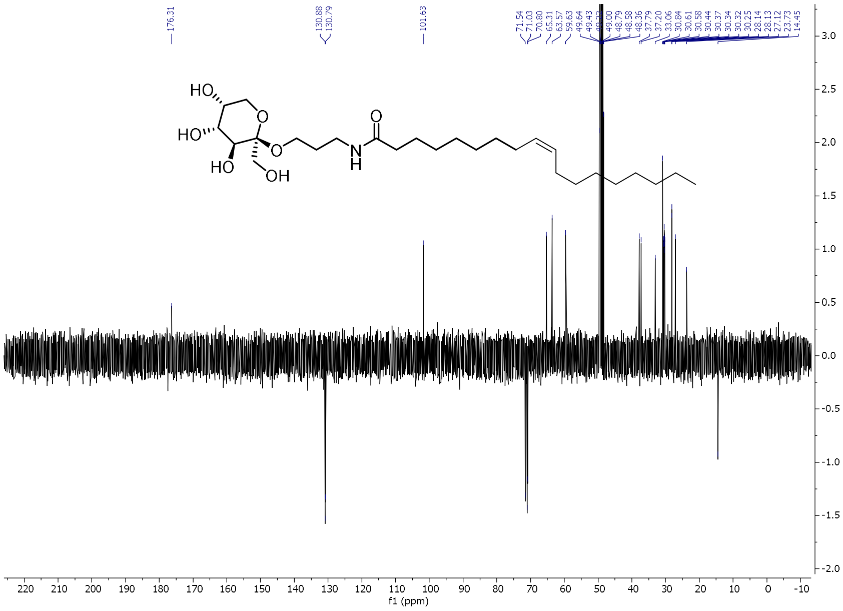

**Figure S21. $^{13}$C-NMR (APT) of 12**

**Correlation Between Surface Tension and the Concentration of Surfactants**

$C_8$-surfactant **8**

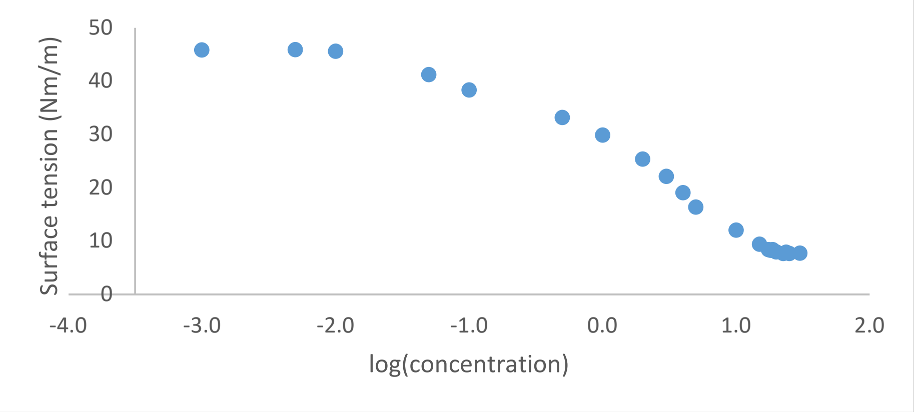


| Concentration (mM) | log[C] | surface tension (mN/m) | Concentration (mM) | log[C] | surface tension (mN/m) |
|---|---|---|---|---|---|
| 0 | | 44,94 | 18,75 | 1,27 | 8,37 |
| 0,001 | -3 | 45,85 | 20 | 1,30 | 7,97 |
| 0,005 | -2,30 | 45,89 | 22,5 | 1,35 | 7,66 |
| 0,01 | -2 | 45,6 | 23,75 | 1,37 | 7,89 |
| 0,05 | -1,30 | 41,22 | 25 | 1,39 | 7,66 |
| 0,1 | -1 | 38,36 | 30 | 1,47 | 7,72 |
| 0,5 | -0,30 | 33,18 | 18,75 | 1,27 | 8,37 |
| 1 | 0 | 29,86 | 20 | 1,30 | 7,97 |
| 2 | 0,30 | 25,37 | 22,5 | 1,35 | 7,66 |
| 3 | 0,477 | 22,15 | 23,75 | 1,37 | 7,89 |
| 4 | 0,602 | 19,09 | 25 | 1,39 | 7,66 |
| 5 | 0,698 | 16,38 | 30 | 1,47 | 7,72 |
| 10 | 1 | 12,03 | | | |
| 15 | 1,17 | 9,42 | | | |
| 17,5 | 1,24 | 8,41 | | | |
| 18,12 | 1,25 | 8,3 | | | |

**Table S7. Effect of $C_8$-surfactant on the interfacial tension between water and pentane**

$C_{10}$-surfactant **9**

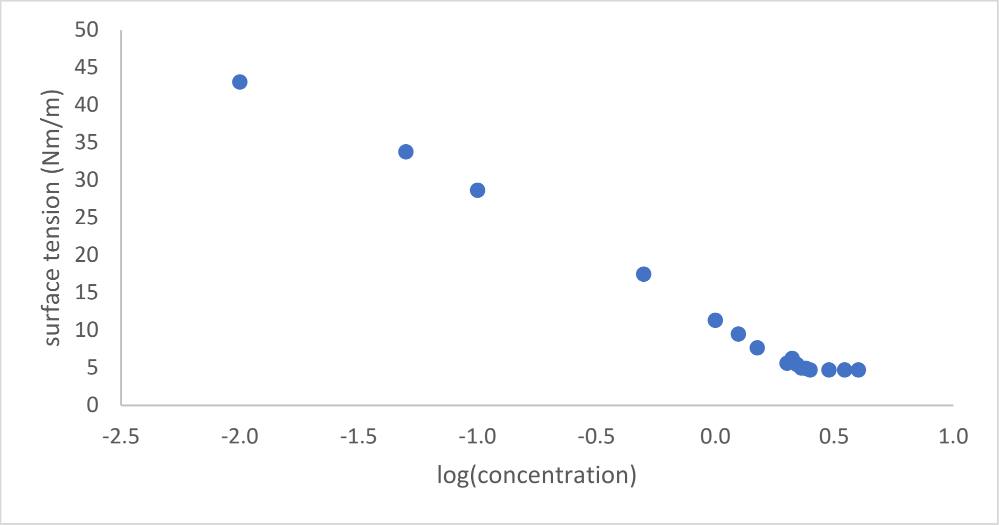


| Concentration (mM) | log[C] | surface tension (mN/m) |
|---|---|---|
| 0 | | 51,68 |
| 0,01 | -2 | 43,11 |
| 0,05 | -1,30 | 33,8 |
| 0,1 | -1 | 28,69 |
| 0,5 | -0,301 | 17,48 |
| 1 | 0 | 11,37 |
| 1,25 | 0,096 | 9,51 |
| 1,5 | 0,176 | 7,67 |
| 2 | 0,301 | 5,63 |
| 2,1 | 0,322 | 6,29 |
| 2,2 | 0,342 | 5,46 |
| 2,3 | 0,361 | 5,02 |
| 2,4 | 0,380 | 4,96 |
| 2,5 | 0,397 | 4,74 |
| 3 | 0,477 | 4,75 |
| 3,5 | 0,544 | 4,73 |
| 4 | 0,602 | 4,73 |

**Table S8. Effect of $C_{10}$-surfactant on the interfacial tension between water and pentane**

**$C_{12}$-surfactant 10**

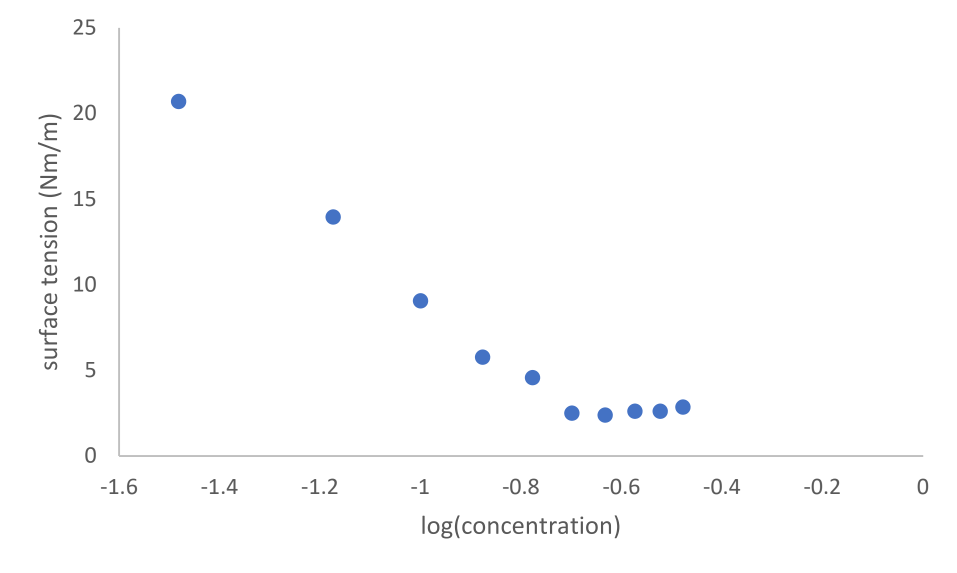


| Concentration (mM) | log[C] | surface tension (mN/m) |
|---|---|---|
| 0 | | 49 |
| 0,033 | -1,48 | 20,7 |
| 0,067 | -1,17 | 13,9 |
| 0,1 | -1 | 9,06 |
| 0,133 | -0,876 | 5,77 |
| 0,167 | -0,777 | 4,57 |
| 0,2 | -0,698 | 2,5 |
| 0,233 | -0,632 | 2,39 |
| 0,267 | -0,573 | 2,61 |
| 0,3 | -0,522 | 2,61 |
| 0,333 | -0,477 | 2,85 |

**Table S9. Effect of $C_{12}$-surfactant on the interfacial tension between water and pentane**

**Decyl-glucopyranoside**

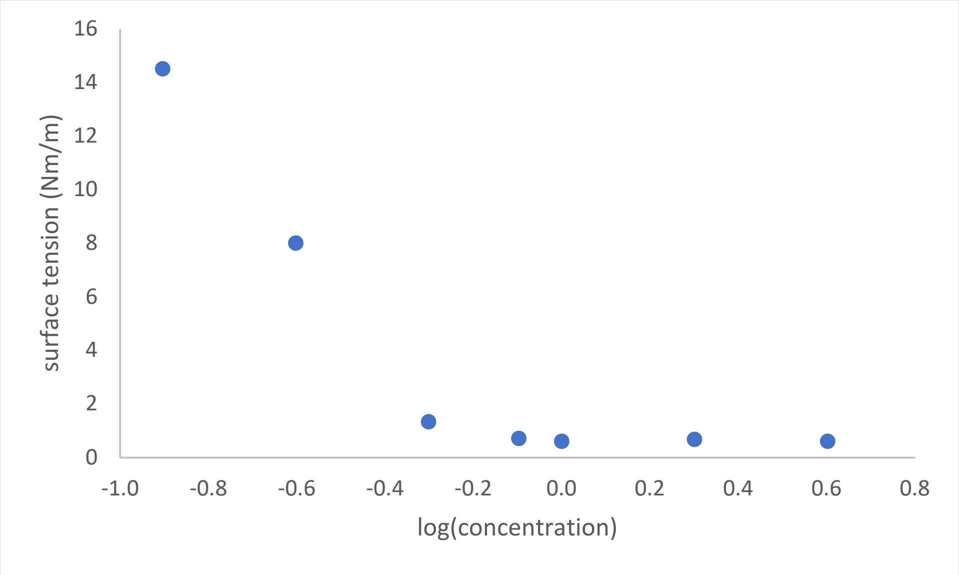


| Concentration (mM) | log[C] | surface tension (mN/m) |
|---|---|---|
| 0 | | 47,19 |
| 0,125 | -0,903 | 14,52 |
| 0,25 | -0,602 | 8,01 |
| 0,5 | -0,301 | 1,35 |
| 0,8 | -0,096 | 0,72 |
| 1 | 0 | 0,62 |
| 2 | 0,301 | 0,69 |
| 4 | 0,602 | 0,61 |

**Table S10. Effect of decyl-glucopyranoside on the interfacial tension between water and pentane**

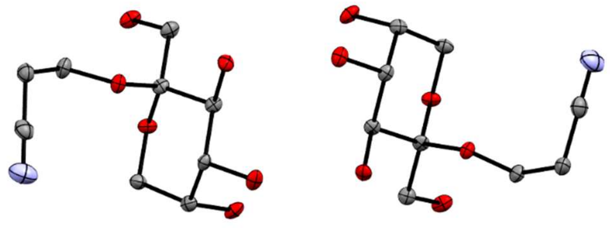

**Figure S22. Molecular structure of compound 2, showing 50% probability ellipsoids. Hydrogen atoms are omitted for clarity. CCDC2310392.**

A single crystal of compound **2** was mounted on top of a cryoloop and transferred into the cold nitrogen stream (100 K) of a Bruker-AXS D8 Venture diffractometer. Data collection and reduction was done using the Bruker software suite APEX3.[i] The final unit cell was obtained from the xyz centroids of 8613 reflections after integration. A multiscan absorption correction was applied, based on the intensities of symmetry-related reflections measured at different angular settings (*SADABS*). The structures were solved by direct methods using *SHELXT*[ii] and refinement of the structure was performed using *SHLELXL*.[iii] The hydrogen atoms were generated by geometrical considerations, constrained to idealised geometries and allowed to ride on their carrier atoms with an isotropic displacement parameter related to the equivalent displacement parameter of their carrier atoms. Crystal data and details on data collection and refinement are presented in Table S11

[i] Bruker, (2016). *APEX3* (v2016.1-0), *SAINT* (Version 8.18C) and *SADABS* (Version 2012/1). Bruker AXS Inc., Madison, Wisconsin, USA.

[ii] Sheldrick, G. M. (2015) *Acta Cryst.* **A71**, 3-8

[iii] Sheldrick, G. M. (2008). *Acta Cryst.* A**64**, 112-122

**Table S11.** Crystallographic data for **2**

| | |
|---|---|
| chem formula | C9 H15 N O6 |
| $M_r$ | 233.22 |
| cryst syst | Monoclinic |
| color, habit | Colourless, needle |
| size (mm) | 0.32 x 0.11 x 0.06 |
| space group | P 21 |
| a (Å) | 5.3370(2) |
| b (Å) | 26.3849(12) |
| c (Å) | 8.0130(3) |
| $\alpha$, deg | 90 |
| $\beta$, deg | 108.970(2) |
| $\gamma$, deg | 90 |
| V (Å$^3$) | 1067.08(8) |
| Z | 4 |
| $\rho_{calc}$, g.cm$^{-3}$ | 1.452 |
| $\mu$(Cu K$\bar{\alpha}$), cm$^{-1}$ | 1.056 |
| F(000) | 496 |
| temp (K) | 100(2) |
| $\theta$ range (deg) | 5.839 – 74.696 |
| data collected (h,k,l) | -6:6, -32:33, -9:9 |
| no. of rflns collected | 16473 |
| no. of indpndt reflns | 4220 |
| observed reflns | 3943 ($F_o \geq 2\ \sigma(F_o)$) |
| R(F) (%) | 3.71 |
| wR($F^2$) (%) | 8.24 |
| GooF | 1.051 |
| Weighting a,b | 0.0288, 0.5211 |
| params refined | 305 |
| restraints | 1 |
| min, max resid dens | -0.263, 0.192 |
